# A Modular Approach to Sensitized Two‐Photon Patterning of Photodegradable Hydrogels

**DOI:** 10.1002/anie.201808908

**Published:** 2018-10-18

**Authors:** Markus Lunzer, Liyang Shi, Orestis G. Andriotis, Peter Gruber, Marica Markovic, Philipp J. Thurner, Dmitri Ossipov, Robert Liska, Aleksandr Ovsianikov

**Affiliations:** ^1^ Institute of Materials Science and Technology TU Wien Getreidemarkt 9/308 1060 Vienna Austria; ^2^ Institute of Applied Synthetic Chemistry TU Wien Getreidemarkt 9/163-MC 1060 Vienna Austria; ^3^ Department of Chemistry-Ångström Laboratory Uppsala University Lägerhyddsvägen 1 751 21 Uppsala Sweden; ^4^ Institute of Lightweight Design and Structural Biomechanics TU Wien Getreidemarkt 9/317 1060 Vienna Austria; ^5^ Department of Biosciences and Nutrition Karolinska Institutet Novum, 141 83 Huddinge Stockholm Sweden; ^6^ Austrian Cluster for Tissue Regeneration Austria

**Keywords:** biomaterials, hyaluronic acid, hydrogels, photochemistry, sensitizers

## Abstract

Photodegradable hydrogels have emerged as useful platforms for research on cell function, tissue engineering, and cell delivery as their physical and chemical properties can be dynamically controlled by the use of light. The photo‐induced degradation of such hydrogel systems is commonly based on the integration of photolabile *o*‐nitrobenzyl derivatives to the hydrogel backbone, because such linkers can be cleaved by means of one‐ and two‐photon absorption. Herein we describe a cytocompatible click‐based hydrogel containing *o*‐nitrobenzyl ester linkages between a hyaluronic acid backbone, which is photodegradable in the presence of cells. It is demonstrated for the first time that by using a cyclic benzylidene ketone‐based small molecule as photosensitizer the efficiency of the two‐photon degradation process can be improved significantly. Biocompatibility of both the improved two‐photon micropatterning process as well as the hydrogel itself is confirmed by cell culture studies.

Hydrogels, crosslinked highly hydrated polymer networks with engineerable properties, are currently utilized in various strategies for tissue engineering, regenerative medicine, and cell delivery, as they permit mimicking the physicochemical properties of the native extracellular matrix (ECM) of soft biological tissues.^1^ Since mammalian cells in vivo are surrounded by a complex three‐dimensional (3D) environment that is remodeled over time, dynamically tunable biomaterials which allow the user to modify the cell surrounding matrix at user‐defined location and time are highly relevant for research on cell and tissue physiology.^2^ A lately emerging, highly elaborated class of such dynamically tunable hydrogels contains photolabile functional groups, which allow for user‐directed real‐time control of the biomaterial's chemical and mechanical properties at positions of interest by irradiation with cytocompatible doses of light.^3^ Such photoresponsive hydrogels have undergone a tremendous evolution in recent years from hydrogels containing photolabile groups on a photostable backbone for guided three‐dimensional cell growth and migration,^4^ over hydrogels with a photodegradable backbone allowing for postgelation control of the physiochemical polymer properties,^5^ to a hydrogel matrix that permits photoreversible spatiotemporal immobilization of proteins,^6^ to mention only a few milestones of this rapid development. The photodegradability of such biomaterials is most commonly enabled by the integration of *o*‐nitrobenzyl (oNB) ester derivatives, which beyond that have been utilized in several other dynamically light triggered materials.^7^ Alternatively, photocleavable hydrogels based on coumarin‐derivatives or ruthenium‐complexes have been reported.^8^ In general, photo‐degradable hydrogels can be photo‐eroded either by the use of UV/Vis light or upon two‐photon excitation.^9^ While the use of UV/Vis light allows for the generation of 2D patterns by applying traditional photolithographic techniques involving masked light,^5^ two‐photon excitation permits the generation of complex 3D patterns by the use of pulsed NIR laser light.^10^ As oNB derivatives typically exhibit relatively low two‐photon absorption cross sections (*δ*_a_) and uncaging action cross sections (*δ*_u_=*δ*_a_ *Q*_u2_, with *Q*_u2_ being the quantum efficiency for uncaging by two‐photon excitation) both in the sub‐GM range,^11^ relatively high laser intensities and long irradiation times are required for photoscission, parameters at which living cells are prone to be damaged.^12^ Moreover, since the laser power required for two‐photon absorption based processes increases with the square root of increasing scanning speed, designing more efficient two‐photon active compounds and processes is a current key challenge in the advancement of high performance multiphoton lithography.^13^ There have been several optimization efforts to promote the efficiency of the oNB photocleavage reaction by varying the number and pattern of substituents on the benzyl group9c, 11a,11c, 14 or by including oNB derivatives into π‐conjugated molecular systems to particularly enhance δ_u_.11a,11c, 15 Alternatively, π‐conjugated compounds have been covalently linked to photocleavable oNB or 7‐nitroindolinyl derivatives in order to sensitize the photoscission reaction by a Förster resonance energy transfer (FRET) process.^16^ Nevertheless, chemical modifications of this kind are labor intensive and usually involve multistep synthesis, whereas certain photolabile oNB based macromer precursors which are known to be cytocompatible are commercially available.^17^ Moreover, the sensitivity of such permanently modified hydrogel systems towards two‐photon excitation is an intrinsic property of the respective precursors that cannot be controlled nor adjusted independently. For these reasons, a modular system in which a small molecule sensitizer is added to the preformed hydrogel in order to promote the two‐photon induced photocleavage reaction would be beneficial.

To this date, the majority of photodegradable hydrogels is based on synthetic polymers as poly(ethylene) glycol (PEG), a material which is biocompatible but offers low cell adhesion and therefore requires the addition of cell‐binding peptide sequences such as Arg‐Gly‐Asp (RGD) or others.^5^, ^18^ Studies that describe the use of other biopolymers including gelatin^19^ or polysaccharides^20^ as backbone for photocleavable hydrogels are still rare. Among natural biopolymers, hyaluronic acid (HA) provides a versatile platform for the synthesis of hydrogel precursors owing to its biocompatibility, chemical modifiability and tunable properties as well as its native biofunctionality due to respective binding receptors such as CD44.^21^ Several studies about light‐controlled postgelation modifications of HA based hydrogels by the use of thiol‐ene chemistry exist, where either hydrogel mechanics have been altered,^22^ or biomolecules have been photo‐patterned into the hydrogels^23^ or both was performed independently.^24^ However, only few of these reports involve the use of photocleavable groups.22c, 23a The utilization of HA as biopolymeric backbone for photodegradable hydrogels is hence an attractive design approach and alternative to currently utilized biomaterials that should definitely be considered.

Herein, we present a modular system that permits the sensitization of the oNB photoscission under two‐photon excitation regime on the example of a photocleavable PEG‐crosslinked HA based hydrogel (PEG‐HA‐SH). We show that by addition of a small molecule exhibiting large two‐photon absorption the efficiency of the oNB‐photoscission can be effectively promoted in a concentration dependent manner and demonstrate the efficacy of this process in the presence of cells. First, a photo‐degradable hydrogel system was designed to be covalently formed and later photo‐patterned based on two orthogonal and biocompatible chemical reactions, namely Michael‐type addition of thiols to acrylates and the photo‐induced cleavage of oNB esters (Figure 1 b, c).^25^

**Figure 1:**
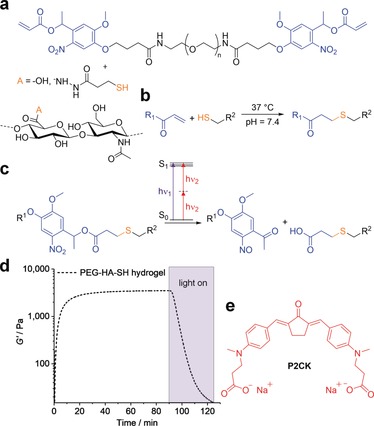
Hydrogel components and reactions used for formation and photo‐degradation of PEG‐HA‐SH hydrogel. a) Molecular structure of photocleavable linker PEG‐(oNB‐A)_2_ and thiol modified hyaluronic acid HA‐SH, which undergo b) crosslinking by Michael‐type thiol‐ene addition at physiological conditions and c) photocleavage of *o*‐nitrobenzyl ester functionalities induced by either UV light or upon two‐photon excitation. d) Network formation and UV/Vis‐induced degradation monitored by oscillatory measurements of the shear storage modulus G′ (320–500 nm, 20 mW cm^−2^). e) Molecular structure of water‐soluble two‐photon sensitizer P2CK.

Hyaluronic acid (HA), a major non‐sulfated glycosaminoglycan component of the ECM,^26^ was chosen as backbone and functionalized with thiol groups to provide the possibility of 3D in situ cell encapsulation by thiol‐ene “click” reaction.^27^ To form the hydrogel, a symmetrical linear poly(ethylene glycol) (PEG, Mw≈6000 Da) based crosslinker containing photo‐labile oNB ester moieties between the PEG chain and terminal acrylate groups was added (PEG‐(oNB‐A)_2_, Figure 1 a).^17^ It is noteworthy, that the Michael‐type thiol‐ene addition is a robust crosslinking reaction for hydrogel formation, which can be performed at cytocompatible conditions in complex formulations including cell culture medium at neutral pH. Thiol modified hyaluronic acid (HA‐SH, Figure 1 a) was synthesized as previously described with a degree of substitution (DS) estimated to be around 50 % by the use of ^1^H NMR spectroscopy (Figure S1, Supporting Information).^28^ To generate a hydrogel formulation, stock solutions of both HA‐SH (18 mg mL^−1^) and PEG‐(oNB‐A)_2_ (150 mg mL^−1^) in cell culture medium were combined with 1:1 stoichiometry of functional groups at neutral pH. Gelation and UV light induced degradation of the networks was examined by irradiation of in situ formed hydrogel samples on a rheometer coupled to a mercury arc lamp. PEG‐HA‐SH hydrogel reached an equilibrium shear storage modulus G′ of 3.7±0.3 kPa within 90 min. Photo‐induced disintegration of the photocleavable hydrogel network was verified by irradiation with UV‐VIS light (320–500 nm, 20 mW cm^−2^) for 35 min leading to a decrease of G′ in the course of illumination (Figure 1 d) but consistency was observed during periods when irradiation was interrupted, confirming that the effect is strictly related to photoirradiation (Figure S5).

We investigated the degradation of the oNB based photocleavable hydrogel by means of multiphoton processing.^29^ In order to improve the efficiency of the oNB ester cleavage under two‐photon excitation regime we hypothesized that this process could be promoted by the addition of a two‐photon active compound, which absorbs light at two‐photon conditions more efficiently and then transfers the energy to the oNB functionalities acting as a two‐photon photosensitizer. To investigate this hypothesis preformed photocleavable hydrogel samples were soaked in solutions of the previously studied water‐soluble and biocompatible cyclic benzylidene ketone‐based two‐photon chromophore P2CK^30^ (Figure 1 e, *δ*_a_≈180 GM) at different concentrations ranging from 0.05 to 0.5 mm in cell culture medium for 5 h. The hydrogel samples were then two‐photon micropatterned using a fs‐pulsed NIR‐laser (800 nm) at a constant writing speed of 200 mm s^−1^ focused through an water immersion objective (32×, 0.85 NA). Parallel channels with rectangular cross sections (300 μm×20 μm×50 μm) were eroded from the edge into the bulk of the hydrogel at different laser powers ranging from 10–100 mW (Figure 2 a). For examination of the fabricated channels the samples were swollen in a solution of fluorescein modified dextran (FITC‐dextran, 1 mg mL^−1^) with an average molecular weight of approximately 2000 kDa (Figure 2 b). Owing to its size the dextran stain only infiltrates the photocleaved areas of the network, but cannot penetrate the bulk hydrogel, permitting visualization of the photo‐eroded channels by confocal laser scanning microscopy (LSM). By these means a distinct relation of the threshold power for the fabrication of an open microchannel by two‐photon degradation in dependence of the P2CK concentration was evaluated.

**Figure 2:**
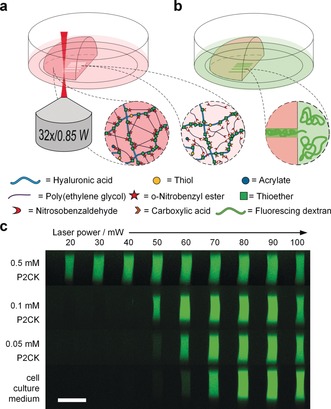
Micro‐channel fabrication by two‐photon degradation of PEG‐HA‐SH hydrogel in presence of two‐photon sensitizer P2CK. a) Schematic illustration of the two‐photon micropatterning of a preformed hydrogel network using a focused NIR‐laser and b) visualization of micro‐channels by soaking in a solution of fluorescent dextran (Mw≈2000 kDa), which owing to its size only infiltrates the micro‐channels. c) Confocal microscopy images displaying orthogonal cross sections (*y*,*z*‐plane) of micro‐channels fabricated with varying laser powers in PEG‐HA‐SH hydrogels after swelling in different solutions of P2CK ranging from 0.05–0.50 mm or pure cell‐culture medium DMEM. Scale bar 50 μm.

In untreated PEG‐HA‐SH hydrogel bright fluorescence could be observed in channels fabricated at laser powers of 70 mW and above. In contrast, with increasing concentration of two‐photon sensitizer P2CK the threshold power decreased accordingly and hence in presence of 0.5 mm P2CK was reduced down to 20 mW (Figure 2 c and Figures S8 and S9). To demonstrate the generality of the presented approach, another photodegradable hydrogel platform based on a thiol‐terminated four‐armed poly(ethylene glycol) (4armPEG‐SH) and PEG‐(oNB‐A)_2_ with 12.5 wt % total macromere content was formed and then two‐photon micropatterned in presence of different concentrations of P2CK as described above. Similar results were achieved in these experiments (Figures S10, S11 and S12). An in situ analysis of the microscale mechanical properties of irradiated sections on PEG‐HA‐SH hydrogel was performed via atomic force microscopy (AFM) cantilever‐based microindentation.^31^ For this purpose, cuboids (A=100×100 μm^2^) with a depth of more than 100 μm were micropatterned into the surface of a hydrogel sample swollen either in PBS or a 0.1 mm solution of P2CK. Again, laser powers from 10–100 mW at a constant writing speed of 200 mm s^−1^ were used. Besides the indentation moduli, the relative heights of the irradiated regions were measured using AFM by referencing to a non‐irradiated position close to the respective region (0 mW). Above a certain laser power the material is eroded and a cavity is formed. The actual depth of the cavities could not be determined by AFM. The laser power thresholds for cavity formation (PBS: 70 mW; 0.1 mm P2CK: 50 mW; Figure 3 a) equal those observed in the experiments in which channels were fabricated (Figure 2 c). However, below the respective threshold the material is incompletely degraded. The decreased crosslinking density results in swelling of the material within the irradiated volume. The degree of swelling increases with the laser power. At 10 mW no swelling is observed in the PBS‐treated PEG‐HA‐SH hydrogel whereas distinct swelling occurs in presence of P2CK.

**Figure 3:**
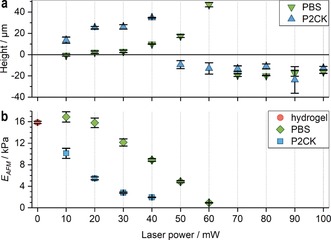
Micro‐mechanical characterization of irradiated sections in PEG‐HA‐SH hydrogel by AFM. a) The relative heights (mean ± s.d.) of two‐photon micropatterned sections in relation to non‐irradiated regions (0 mW) were analyzed by AFM. In PBS soaked hydrogel cavity formation sets in at 70 mW (green inverted triangles), whereas after P2CK‐treatment (0.1 mm) cavities already form at 50 mW (blue triangles). Exposure below the respective threshold laser power leads to incomplete degradation and results in swelling of the irradiated regions. b) The indentation modulus *E*_AFM_ (mean ± s.e.m.) decreases upon irradiation with increasing laser power until the threshold is reached and a cavity is formed.

The indentation modulus, *E*_AFM_, of PEG‐HA‐SH hydrogel is 15.9±0.2 kPa (Figure 3 b; 0 mW; mean ± s.e.m.). In presence of P2CK (0.1 mm) *E*_AFM_ decreases readily upon irradiation with increasing laser power until a cavity is formed at 50 mW. In absence of P2CK a significant decrease of *E*_AFM_ is observed at 30 mW while cavity formation occurs at 70 mW.

While it has been reported that the two‐photon triggered decomposition of photolabile groups can be sensitized by covalently linking two‐photon chromophores to such chemical entities and utilizing a FRET process, multiple synthetic transformations are required therefor.^16^ Moreover, the use of such tandem systems in photocleavable hydrogels would involve the covalent integration of chromophores with unknown bioactivity to the hydrogel backbone. However, by modularly adding a two‐photon active small molecule to the respective hydrogel as solute at an user‐defined point of time the interaction between encapsulated cells and hydrogel matrix is not interfered by chromophores permanently attached to the hydrogel backbone. Additionally, a modular sensitizer system permits the combination of different hydrogel precursors allowing for greater flexibility on achievable materials properties. The sensitized two‐photon patterning was performed at 800 nm, which is a common wavelength of fs‐pulsed Ti:sapphire lasers. Therefore, the developed approach can be used with commercially available multi‐photon microscopes and turn‐key low‐power fs‐pulsed lasers, which became available at a reasonable price in the recent years. Interestingly, as there is no overlap between the P2CK emission and the oNB absorption (Figure S4), a FRET‐based mechanism can clearly be excluded. Besides, the photosensitizing effect of P2CK on the oNB ester cleavage reaction was not observed under UV/Vis light irradiation in respective rheological studies, neither when only P2CK (460 nm), nor when both oNB and P2CK (320–500 nm) were excited (Supporting Information). Hence, the interaction between P2CK and oNB is not trivial and advanced spectroscopic investigations are required to gain an in‐depth understanding of the mechanism.

Nevertheless, we examined the applicability of both PEG‐HA‐SH hydrogel and the P2CK promoted two‐photon degradation process for cell culture. Biocompatibility of P2CK at concentrations below 0.50 mm was verified by metabolic activity assay (Figure S13). Spheroids of GFP‐labeled^32^ hTERT immortalized human adipose‐derived mesenchymal stem cells (ASC/TERT1) were encapsulated in PEG‐HA‐SH hydrogel. 24 h after encapsulation the hydrogel was first soaked in a 0.1 mm solution of P2CK for 5.5 h and then horseshoe‐shaped channels were micropatterned around spheroids at different laser powers ranging from 30–100 mW (Figure 3 and Figure S14 and S15). In addition, two complex spirals were produced and connected to a spheroid at 50 mW and 100 mW (Figure S17, Supporting Information). Within 3 day after micropatterning cell spreading was observed in loops fabricated at laser powers down to 40 mW (Figure 4), indicating that at these conditions the network in the irradiated areas had been sufficiently disintegrated for cells to penetrate. Moreover, after 14 days cells also entered the area irradiated at 30 mW, most likely due to ester hydrolysis of remaining linkages (Figure 4 b).19c On the contrary, in a control sample two‐photon micropatterned without P2CK, cells only entered channels fabricated at laser powers of 60 mW and above within 7 days (Figure S16). Low cytotoxicity of both the hydrogel and the micropatterning process was verified by propidium iodide (PI) staining after 7 days and 14 days showing very few non‐viable cells (red) compared to GFP‐labeled viable cells (green) indicating excellent cell viability (Figure 3 b and Figures S15b and S15c, Supporting Information). Additionally, human osteosarcoma cells (MG‐63) exhibited similar behavior in a preliminary experiment when linear channels had been patterned (Figure S18 and S19).

**Figure 4:**
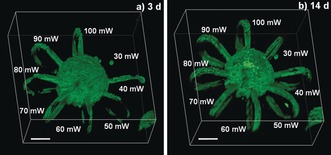
Cell spreading into micro‐loops. GFP‐labeled ASC‐TERT1 spheroids were encapsulated in PEG‐HA‐SH hydrogel. Horseshoe‐shaped micro‐channels (Ø=20 μm×20 μm) were eroded around spheroids with laser powers ranging from 30–100 mW in presence of P2CK (0.1 mm). a) Within 3 days cells entered loops fabricated at laser powers down to 40 mW. b) After 14 days excellent viability was verified by PI staining and cells had also entered the area irradiated at 30 mW. Scale bars 100 μm.

In summary, we present a modular photosensitizing approach for the two‐photon‐induced degradation of a hydrogel based on the new combination of a thiol modified hyaluronic acid and an established PEG‐linker containing photocleavable oNB ester groups. By addition of the two‐photon active molecule P2CK to the preformed hydrogel the threshold power for two‐photon induced photo‐erosion could be successfully decreased. The modular system is biocompatible and can be operated in the presence of encapsulated cells. Through its modularity, this platform is a useful tool for fundamental studies of cell matrix interactions in 3D culture where spatiotemporal control over the cell surrounding matrix by two‐photon micropatterning of photocleavable hydrogels at moderate laser powers is needed.

## Conflict of interest

The authors declare no conflict of interest.

## Supporting information

As a service to our authors and readers, this journal provides supporting information supplied by the authors. Such materials are peer reviewed and may be re‐organized for online delivery, but are not copy‐edited or typeset. Technical support issues arising from supporting information (other than missing files) should be addressed to the authors.

Supplementary
