# Supplementary material for: A Modular Approach to Sensitized Two‐Photon Patterning of Photodegradable Hydrogels
Source: Angew Chem Int Ed Engl. 2018 Oct 18;57(46):15122–7. doi: 10.1002/anie.201808908 (PMC6391948; doi:10.1002/anie.201808908)
Supplement: Supplementary file 1 — Supplementary [file ANIE-57-15122-s001.pdf]

Angewandte Chemie
Eine Zeitschrift der Gesellschaft Deutscher Chemiker

Supporting Information

A Modular Approach to Sensitized Two-Photon Patterning of Photodegradable Hydrogels

*Markus Lunzer, Liyang Shi, Orestis G. Andriotis, Peter Gruber, Marica Markovic, Philipp J. Thurner, Dmitri Ossipov, Robert Liska, and Aleksandr Ovsianikov**

anie_201808908_sm_miscellaneous_information.pdf

S1 Experimental details 2

S1.1 Materials and instrumentation 2

S1.2 Synthesis 2

S1.2.1 Synthesis of thiol-modified hyaluronic acid, HA-SH 2

S1.2.2 Synthesis of 3,3'-Dithiobis(propionic hydrazide), 1 3

S1.2.3 Synthesis of 4-(4-(1-(acryloyloxy)ethyl)-2-methoxy-5-nitrophenoxy)butanoic acid, 3 3

S1.2.4 Synthesis of 4-(4-(1-(acryloyloxy)ethyl)-2-methoxy-5-nitrophenoxy)butanoic acid N-hydroxysuccinimide ester, 4 3

S1.2.5 Coupling of 4-(4-(1-(acryloyloxy)ethyl)-2-methoxy-5-nitrophenoxy)butanoic acid N-hydroxysuccinimide to PEG diamine 4

S1.3 Gel formation of HA-SH hydrogel via Michael thiol-ene reaction 5

S1.4 Gel formation of 4armPEG-SH hydrogel via Michael thiol-ene reaction 5

S1.5 Photo-rheology measurement of photodegradable hydrogels 5

S1.6 Preparation of P2CK solutions 6

S1.7 Two-photon micropatterning of channels in photocleavable hydrogels 6

S1.8 Atomic force microscopy (AFM) indentation testing of two-photon micro-patterned cuboids in PEG-HA-SH hydrogel 6

S1.9 Cell culture and encapsulation of spheroids 7

S1.10 Evaluation of P2CK cytocompatibility 8

S1.11 Two-photon micropatterning, cell viability and spreading 8

S1.12 Functionalization of glass surfaces with methacrylate groups 8

S1.13 Evaluation of optical parameters 9

S2 Supporting Figures 11

S3 References 31

S1 Experimental details

S1.1 Materials and instrumentation

All photosensitive steps were performed under light protection. Unless otherwise stated, chemicals used in the current work were purchased from Sigma-Aldrich and used without further purification. ^{1}H-NMR spectra (400 MHz) were recorded using a Bruker Avance DRX-400 or a JEOL JNM-ECP Series FT-NMR system. Chemical shifts are reported in units of parts per million (ppm) using the residual non-deuterated solvent signal of $CDCl_3$ (δ_H = 7.26 ppm) or D_2O (δ_H = 4.79 ppm) as internal reference. Multiplicities are reported by using the following abbreviations; s: singlet; bs: broad singlet, d: doublet; dd: doublet of doublets; t: triplet; q: quartet; quint: quintet; m: multiplet; *J*: coupling constants in Hertz (Hz). UV-VIS absorption spectra were recorded using a Lambda 35 UV-Vis spectrometer (PerkinElmer). Photoluminescence spectra were collected on a LS 50 fluorescence spectrometer (PerkinElmer).

S1.2 Synthesis

Thiol-modified hyaluronic acid (HA-SH) was synthesized following a previously described procedure.[1] The photocleavable linker PEG-(oNB-A)$_2$ was synthesized according to a modified protocol.[2] The water soluble two-photon dye P2CK was synthesized as described previously.[3]

S1.2.1 Synthesis of thiol-modified hyaluronic acid, HA-SH

Hyaluronic acid (200 mg, M_w 150 kDa, Lifecore Biomedical, HA) was dissolved in deionized water at a concentration of 8 mg ml^{-1}. Dihydrazide linker **1** (Figure S1a) was added to the HA solution at a reagent to HA disaccharide molar ratio of 0.3:1. *N*-hydroxybenzotriazole (HOBt) was separately dissolved in a 1:1 (v/v) mixture of acetonitrile-water at a concentration of 0.2 M and added to the solution of HA at a molar ratio of HOBt to HA disaccharide of 1:1. The pH of the resultant solution was adjusted to 4.7 after which the coupling reaction was initiated by addition of 1-ethyl-3-(3-dimethylaminopropyl) carbodiimide (EDC, 0.5 molar equivalents per HA disaccharide units) to the reaction mixture. The mixture was stirred overnight and then basified to pH 8.5 using 1 M NaOH. *DL*-Dithiothreitol (DTT) was added to the solution in a 5-fold molar excess relative to the estimated amount of disulfide linkages in the HA derivative to ensure the cleavage of disulfide bonds by the reagent. The mixture was stirred overnight. Thereafter the solution was transferred to a dialysis tube (M_w cutoff = 3500 Da) and dialyzed against dilute HCl (pH 3.5) containing 0.1 M NaCl, followed by two dialysis steps against dilute HCl (pH 3.5). After lyophilization of the dialyzed solution thiol-modified HA (181 mg, 80% yield) was obtained as a white solid. HA-SH was estimated to have ~50% of its repetition units

functionalized with thiol groups using ^{1}H-NMR analysis (D_2O) by comparing the integrals of the –C*H₂*C*H₂*SH side chain methylene peaks at 2.58 and 2.73 ppm with the *N*-acetyl moiety of HA at 1.97 ppm (Figure S1b).

S1.2.2 Synthesis of 3,3'-Dithiobis(propionic hydrazide), 1

3,3'-Dithiobis(propionic hydrazide) **1** was synthesized as previously described.[1]

S1.2.3 Synthesis of 4-(4-(1-(acryloyloxy)ethyl)-2-methoxy-5-nitrophenoxy)butanoic acid, 3

4-(4-(1-hydroxyethyl)-2-methoxy-5-nitrophenoxy)butanoic acid **2** (488 mg, 1.63 mmol, CAS: 175281-76-2) and anhydrous DCM (3 mL) were added to a 25 mL round-bottom flask. Triethylamine (661 mg, 6.53 mmol) was added to the round-bottom flask under an argon atmosphere. To the ice cooled solution of starting materials a solution of acryloyl chloride (470 µL, 5.77 mmol) in anhydrous dichloromethane (DCM, 1 mL) was added drop-wise and the combined mixture was then stirred overnight. On the next day the reaction had turned yellow/brown containing white triethylamine•HCl crystals. The reaction mixture was filtered and the organic phase was washed with sodium bicarbonate, 0.1 M aqueous HCl, and concentrated brine. After washing, the solvent was removed by rotary evaporation. An acetone-water mixture (50 mL, 1:1 v/v) was added to the residue and the resulting mixture was stirred overnight. The next day, acetone was removed by rotary evaporation and the remaining water layer was washed four times with DCM. The organic phase was thereafter washed with 0.1 M HCL and brine. DCM was finally evaporated and the product was dried under vacuum overnight, yielding 75% of product **3** (430 mg, 1.22 mmol). ^{1}H-NMR (400 MHz, CD_3Cl, δ): 7.59 (s, 1H, Aromatic-H), 7.00 (s, 1H, Aromatic-H), 6.54 (q, J = 6.4 Hz, 1H,-CH(CH_3)O-), 6.44 (dd, J_{vic} = 17.3 Hz, J_{gem} = 1.3 Hz, 1H, -CH=CHH), 6.17 (dd, J_{vic} = 17.3, 10.4 Hz, 1H, -CH=CHH), 5.87 (dd, J_{vic} = 10.4 Hz, J_{gem} = 1.3 Hz, 1H, -CH=CHH), 4.12 (t, J = 6.1 Hz, 2H, 4-CH_2 of butanoate), 3.92 (s, 3H, CH_3O-), 2.61 (t, J = 7.1 Hz, 2H, 2-CH_2 of butanoate), 2.19 (quint, J = 6.6 Hz, 2H, 3-CH_2 of butanoate), 1.66 (d, J = 6.4 Hz, 3H, -CH(CH_3)O-).

S1.2.4 Synthesis of 4-(4-(1-(acryloyloxy)ethyl)-2-methoxy-5-nitrophenoxy)butanoic acid N-hydroxysuccinimide ester, 4

4-(4-(1-(acryloyloxy)ethyl)-2-methoxy-5-nitrophenoxy)butanoic acid **3** (79.6 mg, 0.225 mmol, CAS: 175281-76-2), *N*-hydroxysuccinimide (29.4 mg, 0.27 mmol) and EDC (43.4 mg, 0.28 mmol) were added to a 25 mL round bottom flask and it was purged with argon. Anhydrous DCM (4 mL) was then added and the reaction mixture was stirred overnight at room temperature. It was then washed three times with concentrated brine and dried with magnesium sulfate. The solvent was evaporated and the obtained residue was dried under vacuum overnight, yielding 68% of product **4** (69 mg, 0.153 mmol). ^{1}H-NMR (400 MHz, CD_3Cl, δ): 7.60 (s, 1H, Aromatic-H), 7.01 (s, 1H, Aromatic-H), 6.54 (q, J = 6.2 Hz, 1H, -CH(CH_3)O-),

6.43 (dd, J_{vic} = 17.3 Hz, J_{gem} = 1.3 Hz, 1H, -CH=C<u>H</u>H), 6.17 (dd, J_{vic} = 17.3, 10.5 Hz, 1H, -C<u>H</u>=CHH,), 5.87 (dd, J_{vic} = 10.5 Hz, J_{gem} = 1.3 Hz, 1H, -CH=CH<u>H</u>), 4.16 (t, *J* = 6.0 Hz, 2H, 4-C$\underline{H}_2$ of butanoate), 3.93 (s, 3H, C$\underline{H}_3$O-), 2.90-2.82 (m, 6H, NHS and 2-C$\underline{H}_2$ of butanoate), 2.28 (quint, *J* = 6.7 Hz, 2H, 3-C$\underline{H}_2$ of butanoate), 1.66 (d, *J* = 6.4 Hz, 3H, -CH(C$\underline{H}_3$)O-).

S1.2.5 Coupling of 4-(4-(1-(acryloyloxy)ethyl)-2-methoxy-5-nitrophenoxy)butanoic acid N-hydroxysuccinimide to PEG diamine

4-(4-(1-(acryloyloxy)ethyl)-2-methoxy-5-nitrophenoxy)butanoic acid *N*-hydroxysuccin-imide ester **4** (117 mg, 0.26 mmol) and PEG diamine (600 mg, 0.10 mmol, M_n = 6,000 Da, ABCR GmbH) were added to a 50 mL round bottom flask. The flask was purged with argon and 13 mL of anhydrous DCM was added to the flask. Triethylamine (2 µL, 0.02 mmol) was added to the reaction mixture and it was stirred overnight. After 24 h it was precipitated in cold diethyl ether. The precipitate was centrifuged (1400 rcf, 4 °C, 20 min), washed again and then dried in vacuum finally yielding 93% (624 mg, 0.93 mmol) of PEG-(oNB-A)$_2$ as a white powder.

^{1}H-NMR (400 MHz, CD_3Cl, δ): 7.58 (s, 2H, Aromatic-<u>H</u>), 7.00 (s, 2H, Aromatic-<u>H</u>), 6.52 (q, *J* = 6.4 Hz, 2H, -C<u>H</u>(CH$_3$)O-), 6.42 (dd, J_{vic} = 17.3 Hz, J_{gem} = 1.2 Hz, 2H, -CH=C<u>H</u>H), 6.25 (bs, 2H, N<u>H</u>), 6.16 (dd, J_{vic} = 17.3, 10.5 Hz, 2H, -C<u>H</u>=CHH), 5.86 (dd, J_{vic} = 10.5 Hz, J_{gem} = 1.2 Hz, 2H, -CH=CH<u>H</u>), 4.10 (t, *J* = 6.2 Hz, 4H, 4-C$\underline{H}_2$ of butanamide), 3.93 (s, 6H, C$\underline{H}_3$O-), 3.82-3.44 (m, ~616H, PEG diamine), 2.40 (t, *J* = 7.3 Hz, 4H, 2-C$\underline{H}_2$ of butanamide), 2.19 (quint, *J* = 6.6 Hz, 4H, 3-C$\underline{H}_2$ of butanamide), 1.65 (d, *J* = 6.4 Hz, 6H, -CH(C$\underline{H}_3$)O-).

S1.3 Gel formation of PEG-HA-SH hydrogel via Michael thiol-ene reaction

HA-SH was dissolved in Dulbecco's modified Eagle's medium (DMEM) or Endothelial cell Growth Medium (EGM-2, Lonza) and neutralized with aqueous NaOH (0.1 M) to form an 18 mg ml^{-1} solution. PEG-(oNB-A)$_2$ was dissolved in PBS giving a solution of 150 mg ml^{-1}. The solutions were combined with 1:1 stoichiometry of functional groups at 6.7 wt% total macromer concentration and used immediately. Hydrogels based on HA-SH with different DS were prepared analog but resulting in a different total macromer concentration (HA-SH DS = 40%: 5.5 wt%).

S1.4 Gel formation of 4armPEG-SH hydrogel via Michael thiol-ene reaction

A photodegradable hydrogel based on PEG-(oNB-A)$_2$ and thiol-terminated four-armed poly(ethylene glycol) (4armPEG-SH) was fabricated as previously reported by Tibbitt *et al*.[4] 4armPEG-SH (M_w ~5,000 kDa, Sigma-Aldrich) and PEG-(oNB-A)$_2$ were separately dissolved in deionized water to form 20 wt% stock solutions. The solutions were combined with 1:1 stoichiometry of functional groups at 12.5 wt% total macromer concentration. In a typical example, 7.8 µL of 4armPEG-SH solution, 20.9 µL of PEG-(oNB-A)$_2$ solution and 10.3 µL of PBS were combined. To accelerated hydrogel formation, 1 µL of NaOH (0.1 mM) was added and the hydrogel precursor solution was immediately used.

S1.5 Photo-rheology measurement of photodegradable hydrogels

The time required for network formation was estimated by measuring the shear storage modulus G' using a MCR-302 WESP rheometer with a P-PTD 200/GL peltier glass plate and a PP25 measuring system (Anton-Paar). Hydrogel samples were prepared as described in the above sections. For control experiments involving P2CK the linker PEG-(oNB-A)$_2$ was dissolved in an adequate amount of P2CK solution (1.0 mM) and PBS so that the resulting hydrogel formulation contained 0.1 mM P2CK. Immediately after mixing of the components 55 µl of the solution were placed at the center of the glass plate. The measurements were conducted at 20 °C using a plate-plate measuring system (Ø = 25 mm, gap size = 50 µm). The formulations were sheered with a strain of 1% and a frequency of 1 Hz. Gelation was assumed to be complete when G' reached a plateau. Photo-degradation of hydrogel samples was induced by UV-irradiation projected *via* a waveguide (35 min, 320–500 nm, ~20 mW cm^{-2} at the measuring platform) from the underside of the glass plate using an OmniCure® S2000 Spot UV Light Curing System (Excelitas Technologies).[5] Alternatively, an OmniCure® LX400 LED UV Spot Curing System with a 460 nm LED head was used (Excelitas Technologies) with a specific power of ~17 mW cm^{-2} at the measuring platform.

S1.6 Preparation of P2CK solutions

Solutions of P2CK at concentrations ranging from 1.00 to 0.01 mM were freshly prepared before each experiment by dilution of a stock solution in PBS (10 mM) with either adequate cell culture medium or PBS.

S1.7 Two-photon micropatterning of channels in photocleavable hydrogels

Aliquots (40 µl) of the mixed PEG-HA-SH hydrogel precursor solutions were drop casted onto methacrylized glass-bottom µ-dishes (35 mm, Ibidi GmbH, Germany) and allowed to gel at room temperature for 90 min in accordance to the gelation duration observed by rheological measurements. The solidified hydrogel droplets were immersed in either DMEM or solutions of P2CK and swollen for ~5 h at room temperature before one half of each sample was cut away using a scalpel to generate a sharp edge. Aliquots (27 µl) of the mixed 4armPEG-SH hydrogel precursor solutions were formed in cylindrical silicon molds (d = 6 mm, h = 0.7 mm) between glass-bottom µ-dishes and hydrophobized cover glass. After 40 min the molds were carefully removed and the 4armPEG-SH hydrogels were swollen in PBS overnight before they were treated with P2CK as described above.

Microfabrication was performed by means of two-photon degradation. Details of the experimental setup have been reported previously.[6] Briefly, the setup is based on a femtosecond laser (MaiTai DeepSee, Spectra Physics) operating at 800 nm, with a pulse length of 70 fs after the objective (C-Achroplan 32x/0.85 W, ZEISS). Parallel channels with rectangular cross sections (l = 300 µm, A = 20 µm x 50 µm) were fabricated starting from the edge into the bulk of the hydrogel at a height of ~60 µm (~200 µm in case of 4armPEG-SH hydrogels) above the glass plate with varied mean laser power per channel (10–100 mW, 10 mW steps) and a constant scanning speed of 200 mm/s (hatch distance: 0.1 µm, z-layer distance: 0.5 µm). The samples were washed with PBS twice and then soaked in a solution of FITC–dextran (1 mg ml^{-1}, M_w ~2,000 kDa, TdB Consultancy AB, Sweden) in PBS at room temperature overnight. Channels were visualized by laser scanning microscopy (LSM 700, ZEISS).

S1.8 Atomic force microscopy (AFM) indentation testing of two-photon micropatterned cuboids in PEG-HA-SH hydrogel

A droplet (10 µL) of PEG-HA-SH hydrogel was prepared in a cylindrical mold (d = 6 mm, h = 0.2 mm) on methacrylized glass bottom µ-dishes. To ensure a smooth surface no lid was used for molding. After 90 min the mold was removed and the hydrogel sample was swollen in PBS for 2.5 h. Cuboids (A = 100 x 100 $µm^2$) with an approximate depth of 100–150 µm were two-photon micropatterned from the upper surface into the hydrogel starting above the surface. The mean laser power was varied per cuboid (10–100 mW, 10 mW steps) and a constant scanning speed of 200 mm/s (hatch distance: 0.1 µm, z-layer distance: 0.5 µm) was used.

Subsequently, PBS was exchanged for a 0.1 mM solution of P2CK in PBS and the sample was maintained at room temperature for 1 h. Again, cuboids were micropatterned into the hydrogel at the same parameters as before. Thereafter, the hydrogel was swollen in PBS overnight. A NanoWizard® ULTRA SpeedA AFM system (JPK Instruments AG, Germany) equipped with an inverted optical microscope (Axio Observer.D1, ZEISS) was used for AFM experiments. The micromechanical assessment was performed via AFM cantilever-based microindentation experiments using a silicon nitride rectangular cantilever (0.0196 N m^{-1} measured spring constant; MSNL, Bruker) equipped with a colloidal probe of 4.75 µm in radius. The thermal noise method[7] was used to calibrate the cantilever spring constant. The deflection sensitivity was obtained by performing 16 force measurements on the glass surface next to the sample.[8] After calibration, the cantilever was fabricated with the colloidal probe[9] and its diameter was measured as previously described.[8] In total, 50-60 force curves were recorded at 1 nN maximum load and 2 µm s^{-1} of z-displacement speed per irradiated section. The Oliver-Pharr method was used to analyze the force curves and estimate the indentation modulus, as previously described, in a custom MATLAB script (v R2015b, MathWorks, USA).[8] For the analysis, the Poisson's ratio of the hydrogel was taken as 0.5. The relative height measurement results are presented as mean ± standard deviation. The indentation modulus results are presented as mean ± standard error of the mean.

S1.9 Cell culture and encapsulation spheroids

hTERT immortalized human adipose-derived mesenchymal stem cell line ASC/TERT1 (Evercyte GmbH, Austria) was retrovirally transfected with green fluorescent protein (GFP) to obtain stably transfected green labeled cells following a previously established protocol.[10] ASC/TERT1 were cultured in EGM-2 media supplemented with 10% fetal bovine serum (Lonza). MG-63 cells (Sigma-Aldrich) were cultured in DMEM supplemented with 10% fetal bovine serum and 1% penicillin/streptomycin (Lonza).

Cells were maintained in an incubator (5% CO_2, 37 °C) and the medium was exchanged every second day. Cell spheroids were formed in 2% agarose molds (MicroTissues® 3D Petri Dish®, Sigma-Aldrich) according to the manufacturer's instructions. In order to generate spheroids with a diameter of 200 µm 81,000 cells were seeded per micromold corresponding to 1000 cells per spheroid. Cells were incubated for 24 to 48 h. For ASC/TERT1 encapsulation 0.90 mM RGDS (Bachem AG, Switzerland) was added to the PEG-$(oNB\text{-}A)_2$ stock solution and reacted for 10 min. Spheroids of two molds were combined and collected before they were resuspended in 100 µl of premixed PEG-HA-SH hydrogel precursor solution (ASC/TERT1: with RGDS (0.29 mM); MG-63s: HA-SH with DS = 40%) by cautious pipetting (~5x). Aliquots of the suspension (30 µl) were drop casted onto methacrylized glass bottom µ-dishes. After short reaction time (~10 min) the samples were maintained in the incubator for 90 min to allow hydrogel formation to complete before cell culture media was added (2.5 ml).

S1.10 Evaluation of P2CK cytocompatibility

The cytocompatibility of two-photon sensitizer P2CK was evaluated using PrestoBlue® cell viability reagent (Thermo Fisher Scientific). Two 96-well plates were seeded with 5,000 cells per well and maintained in the incubator overnight to allow cells to attach. Next day, cells were incubated with 100 µL of different dilutions of P2CK (1.00, 0.50, 0.10, 0.05 and 0.01 mM) for 3 h and 24 h respectively. Both plates contained wells with non-treated cells as control samples. After the respective incubation period, the cell culture medium was exchanged twice to remove residues of P2CK and cell viability was evaluated. Resazurin-based reagent PrestoBlue® was diluted 1:10 with medium and 100 µL were applied per well and incubated for 60 min. Because of the reducing environment of viable cells, this reagent is transformed and turns red, becoming highly fluorescent. The fluorescence was measured with a plate reader (Synergy BioTek, excitation 560 nm, emission 590 nm). After correction for background fluorescence, the signals of the cells exposed to different concentrations of P2CK were compared to each other and to the controls (non-stimulated cells) by one-way ANOVA and Dunnett's multiple comparisons test. The metabolic activity of the 3 h control was assumed to be 100%. Statistical data evaluation was performed using the software package GraphPad Prism 5 (GraphPad Software, USA) and Excel 2016 (MS Office, Microsoft).

S1.11 Two-photon micropatterning, cell viability and spreading

PEG-HA-SH hydrogel with encapsulated spheroids was soaked in a solution of P2CK (0.1 mM in adequate medium) for 5.5 h (MG-63s: 1 h). Thereafter, channels were eroded with varying laser intensities around spheroids in a star-shaped manner into the bulk hydrogel at constant writing speed of 200 mm s^{-1}. The laser power was increased clockwise (30–100 mW, 10 mW steps). All other parameters were kept identical as described above. The samples were washed with medium twice and then incubated (5% CO_2, 37 °C) in cell culture medium overnight. LIVE/DEAD viability assay (Life Technology) was used to monitor MG-63 viability and spreading. Samples were stained for 20 min at 37 °C with a solution of calcein AM (0.2 µM) and propidium iodide (0.6 µM, PI) in PBS and thereafter imaged by the use of LSM. For better evaluation of ASC/TERT1 viability additional PI staining was applied on day 7 and 14.

S1.12 Functionalization of glass surfaces

Glass bottom µ-dishes were pre-treated in a plasma cleaner (PDC-002, Harrick Plasma, USA) for 10 min. A methacrylization solution was prepared by combining deionated water (50% v/v), ethanol (48% v/v), glacial acetic acid (0.3% v/v) and 3-(trimethoxysilyl)propyl methacrylate (2% v/v). The solution was stirred for 15 min before aliquots (~1 mL) were pipetted into the µ-dishes. After 30 min of surface treatment, µ-dishes were rinsed with deionated water twice and then dried in a drying chamber (50 °C) for ~1 h. Cover glass were hydrophobized in a similar manner using n-octadecyltrimethoxysilane (ABCR GmbH, Germany)

S1.13 Evaluation of optical parameters

To fully describe an experiment with multiphoton activation, parameters such as laser power after the objective, numerical aperture of the objective, wavelength, scanning speed, hatch and z-layer spacing need to be stated. Unfortunately, in many publications not all of these parameters are presented, which makes it very difficult to compare experiments. Therefore, we would like to introduce this parameter set as a guideline for future publications.

The peak intensity I_{Peak} is an important factor, because it allows the direct comparison of two different multiphoton polymerization systems. As an example, objectives with different numerical aperture NA perform the same, if the peak intensity is the same, and the average power has been adjusted correspondingly. The peak intensity is obtained by dividing peak power P_{Peak} by the beam area and multiplied with the correction factor of 2 for a Gaussian beam

$$I_{Peak} = \frac{2\,P_{Peak}}{\pi {\omega_0}^2} \tag{1}$$

Whereas the peak power of a Gaussian beam is

$$P_{Peak} = 0.94\,\frac{E_P}{\tau_P} \tag{2}$$

with the pulse length τ_P and the pulse energy E_P, which is the average laser power divided by the laser repetition rate. The Gaussian beam waist radius ω_0 of a well-corrected optical system such as a microscope objective with a given numerical aperture at the wavelength λ is estimated by

$$\omega_0 \cong \frac{\lambda}{\pi\,\mathrm{NA}} \tag{3}$$

The peak intensity for the parameters used in this work (λ: 800 nm; repetition rate: 80 MHz; τ_P: 70 fs; NA: 0.85) per mW is:

$$I_{Peak} = 119\ \frac{\mathrm{GW}}{\mathrm{cm}^2}$$

Further noteworthy parameters are the voxel dimensions. The diffraction-limited lateral (ω_{xy}) and axial (ω_z) e^{-1} radii of the squared illumination point spread function (IPSF^2) can be calculated as shown in the work of Zipfel et al.[11]

$$\omega_{xy} = \begin{cases} \dfrac{0.320\,\lambda}{\sqrt{2}\,\mathrm{NA}} & \mathrm{NA} \leq 0.7 \\[2ex] \dfrac{0.325\,\lambda}{\sqrt{2}\,\mathrm{NA}^{0.91}} & \mathrm{NA} > 0.7 \end{cases} \tag{4}$$

$$\omega_z = \frac{0.532\,\lambda}{\sqrt{2}}\left[\frac{1}{n-\sqrt{n^2-\mathrm{NA}^2}}\right] \tag{5}$$

Whereas full width at half maximum (FWHM) can be obtained by multiplying ω_{xy} with $2\sqrt{\ln 2}$ and for the e^{-2} radius with $\sqrt{2}$ respectively. The FWHM is a good estimation for the real size of a voxel, however by fine tuning the laser power very thin lines can be produced. The following FWHM values for the parameters used in this work are obtained:

$$\mathrm{FWHM}_{xy} = 0.35\ \mu\mathrm{m} \qquad \mathrm{FWHM}_{z} = 1.63\ \mu\mathrm{m}$$

S2 Supporting Figures

a

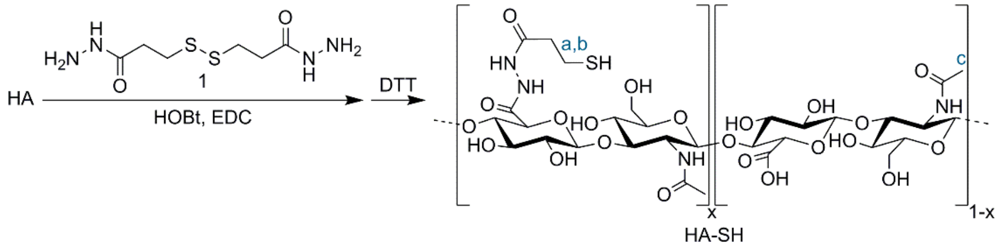

b

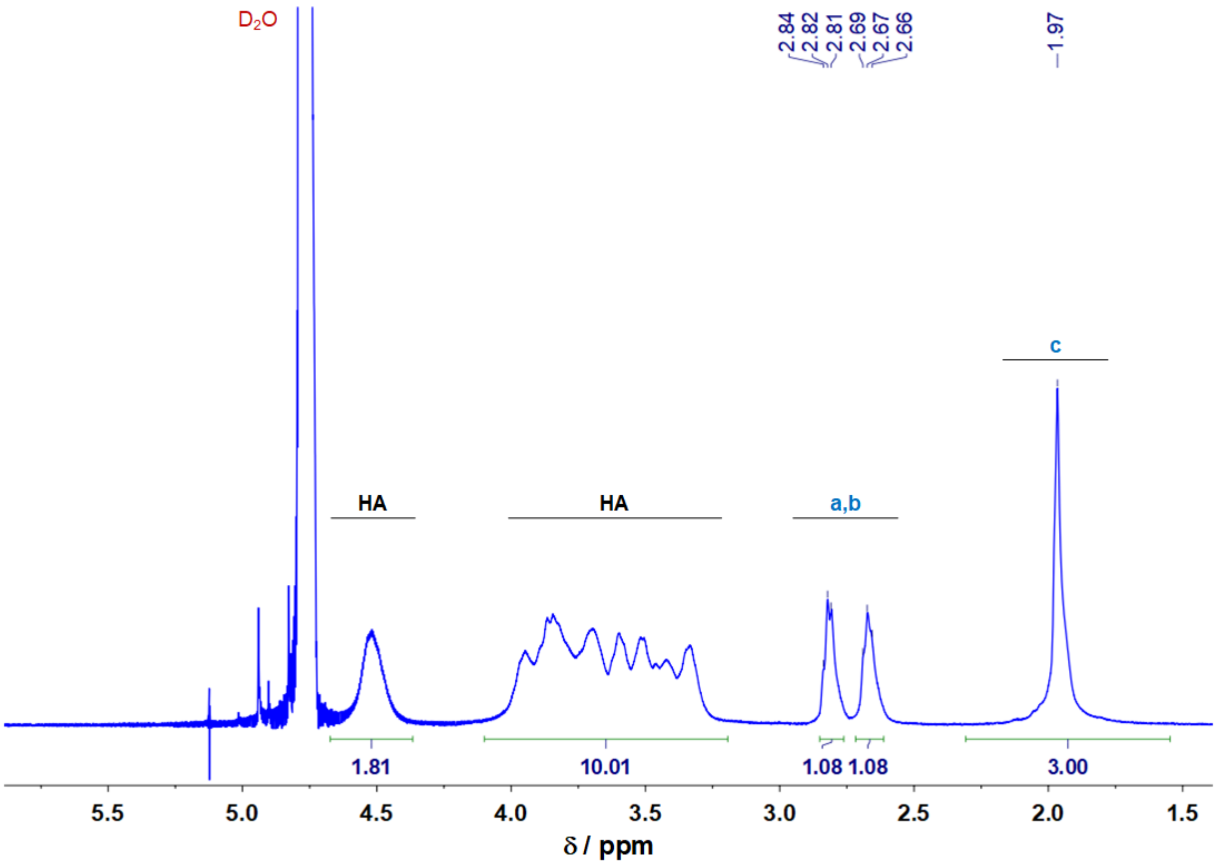

Figure S1. Synthesis route and characterization of HA-SH.

a) Hyaluronic acid was reacted with 3,3'-dithiobis(propionic hydrazide) **1** in presence of *N*-hydroxybenzotriazole (HOBt) and 1-ethyl-3-(3-dimethylaminopropyl) carbodiimide (EDC) and subsequently reduced to HA-SH using *D,L*-dithiothreitol (DTT). b) ^{1}H-NMR spectra for HA-SH. The corresponding groups of each peak are shown in the plot. The degree of thiol functionalisation of HA-SH (DS) was estimated to be ~50% via ^{1}H-NMR analysis. The integral of the methyl peak of the acetyl group **c** was utilized as reference for the determination of the DS by calculating its relative ratio to that of the methylene groups in α,β-position to the thiol group **a,b**.

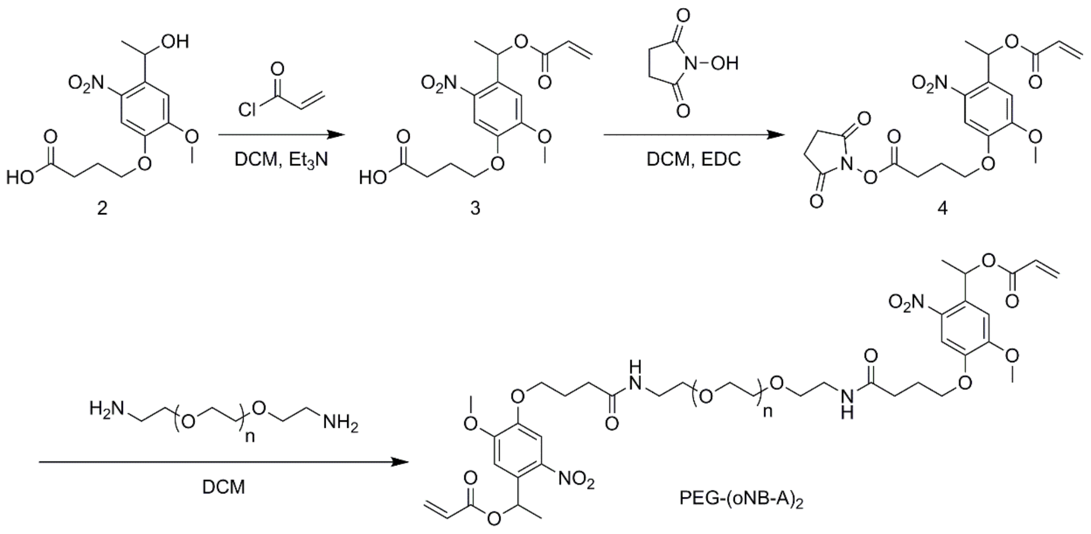

Figure S2. Synthesis route towards photocleavable linker PEG-(*o*NB-A)$_2$.

The photocleavable linker was synthesized following a modified version of a previously described protocol.[2] 1-Nitrobenzyl alcohol **2** was reacted with acryloyl chloride in presence of trimethylamine to 1-nitrobenzyl ester **3**. After activation of ester **3** by conversion into the corresponding *N*-hydroxysuccinimidyl ester **4** using EDC chemistry it was coupled to PEG diamine (M_n = 6,000 Da) to give the photocleavable linker PEG-(oNB-A)$_2$.

a

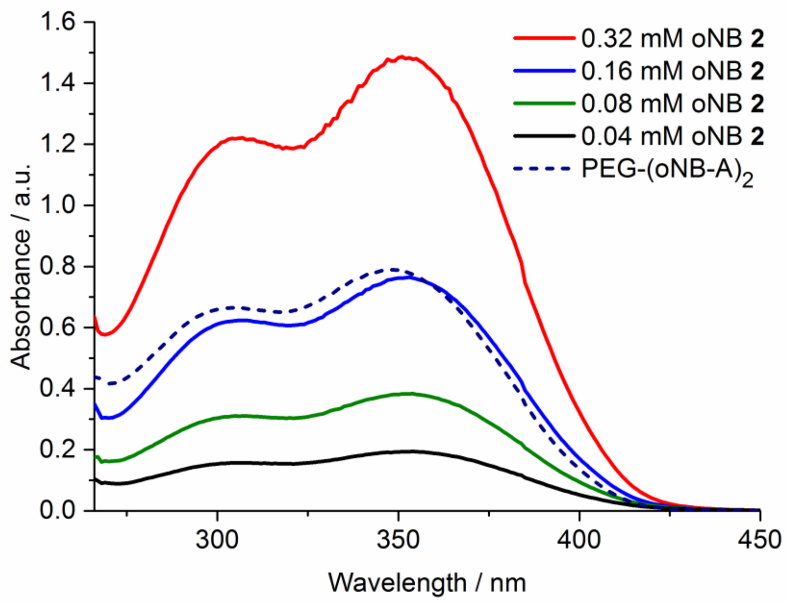

b

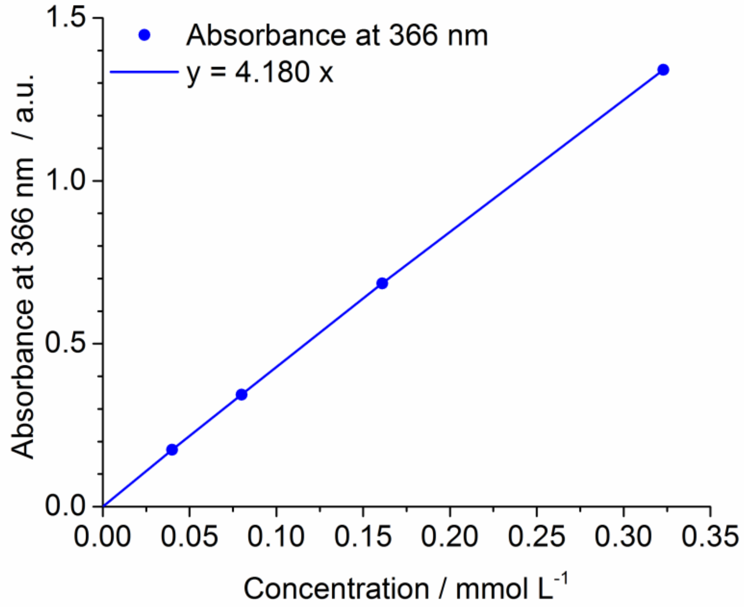

Figure S3. Characterization of the PEG linker by UV-Vis spectrometry.

Modification of PEG diamine (M_n = 6,000 Da) with photocleavable *ortho*-nitrobenzyl groups was verified by UV-Vis spectroscopy. 4-(4-(1-Hydroxyethyl)-2-methoxy-5-nitrophenoxy) butanoic acid **2** in dimethylformamide (DMF) was used as reference compound. a) UV-Vis spectra of **2** were recorded at concentrations of 0.323 mM (red curve), 0.161 mM (blue curve), 0.080 mM (green curve), and 0.040 mM (black curve). Moreover, a solution of PEG-(*o*NB-A)$_2$ at a concentration of 0.5 mg ml^{-1} in DMF was measured (blue dashed curve). b) Based on the

above spectra, a calibration curve was fitted for the absorption of **2** at 336 nm giving an equation for the determination of the concentration of *ortho*-nitrobenzyl (*o*NB) moieties according to Beer–Lambert's law $A_{\lambda} = \varepsilon_{\lambda}\, d\, c$ (where *A* is the optical density, ε_{λ} the molar extinction coefficient in L mol^{-1} cm^{-1}, *d* the path length in cm and *c* the concentration in mol L^{-1}): $A_{336nm} = 4.180\, c$. Considering the theoretical molecular weight of PEG-(*o*NB-A)$_2$ being ~6670 g mol^{-1} the *o*NB concentration of the linker solution is expected to be ~0.150 mM. The absorption of the linker solution measured at 336 nm was equal to 0.745 which corresponds to 0.178 mM concentration of *o*NB moieties according to the derived equation. The deviation of the measured from the expected value (~19%) can be attributed to uncertainties in molecular weight determination of PEG diamine and matrix effects.

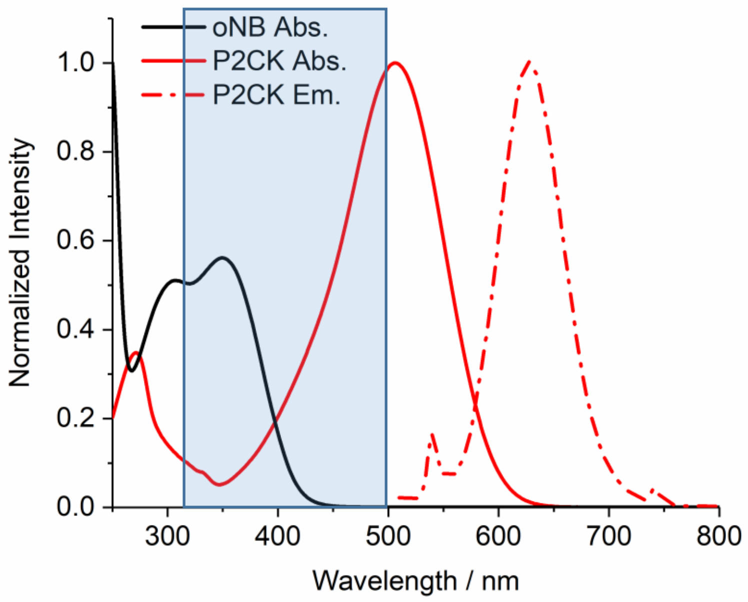

Figure S4: One-photon spectroscopy of P2CK and oNB moiety **2**.

The linear absorption spectrum of oNB moiety **2** (190 µM) as well as the absorption and emission spectra of P2CK (11 µM) were measured in PBS. The absorption spectra of P2CK (red line) and oNB moiety **2** (black line) overlap with the UV-light source used in the photorheology experiments (320–500 nm, blue area) whereas only P2CK is excited by the 460 nm LED. However, the emission spectrum of P2CK (red dotted line) does not overlap with the absorption spectrum of oNB **2**. Hence, a FRET-based sensitization mechanism can be excluded.

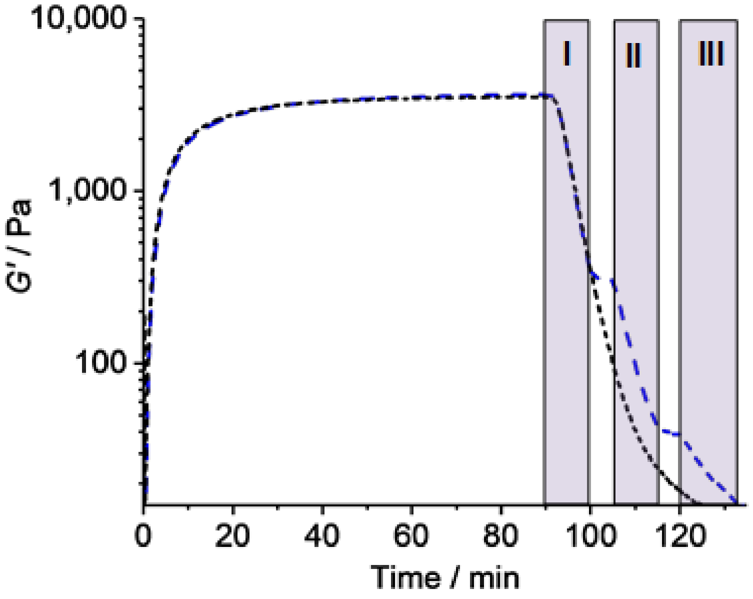

Figure S5. Rheology measurement of the shear storage modulus G'.

Optically thin photocleavable hydrogel networks (50 µm) have been formed in situ between a peltier glass plate and a measuring plate (25 mm) on a photorheometer (MCR-302 WESP, Anton-Paar). After constant G' was observed (90 min) photo-induced degradation of hydrogel networks was demonstrated by irradiation with shuttered UV-VIS light (I, II, III, 320–500 nm, 20 mW cm^{-2}, blue dashed line) leading to a decrease of G' in the course of illumination but consistency during periods when irradiation was interrupted, confirming that the effect is strictly related to photoirradiation. In contrast, when the network was continuously irradiated a stepless decline of G' occurred (black dotted line).

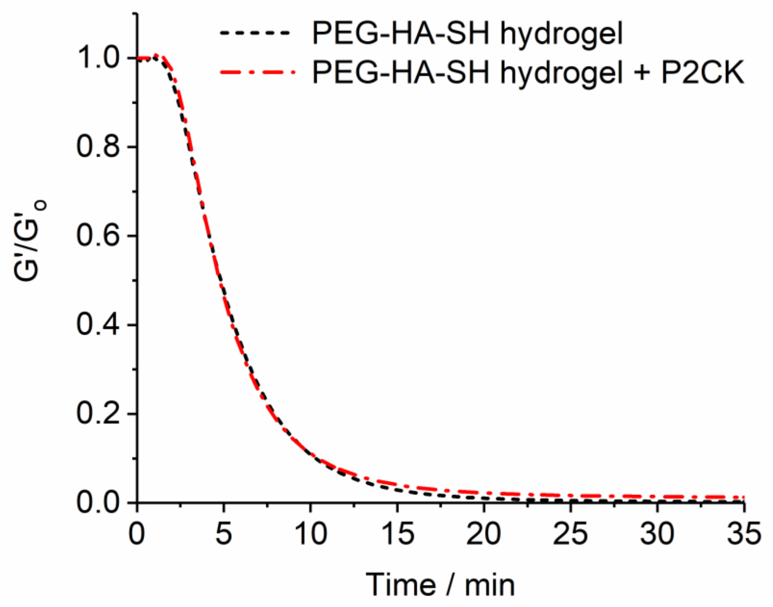

Figure S6. UV-VIS degradation of PEG-HA-SH hydrogel in presence of P2CK and without. Optically thin hydrogels (50 µm) were in situ formed on the measuring platform of a photorheometer either in presence of P2CK (0.1 mM) or without. No pronounced effect of P2CK on the one-photon induced degradation (320–500 nm, ~20 mW cm^{-2}) of the photocleavable hydrogel networks could be observed.

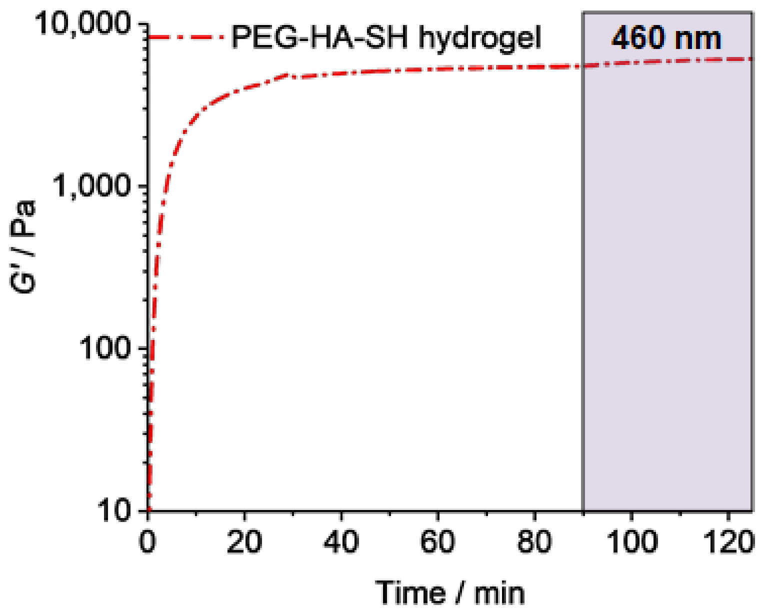

Figure S7. Irradiation of PEG-HA-SH hydrogel in presence of P2CK at 460 nm.

Optically thin hydrogels (50 µm) were in situ formed on the measuring platform of a photorheometer in presence of P2CK (0.1 mM). The hydrogel was irradiated at 460 nm using an LED (~17 mW cm^{-2}, 35 min). While P2CK is specifically excited at 460 nm, this wavelength lies outside the absorption range of oNB. No decrease of G' could be observed. A slight increase of G' in the course of irradiation can be attributed to polymerization of unreacted pending groups due to weak initiation properties of typical two-photon initiators.[12]

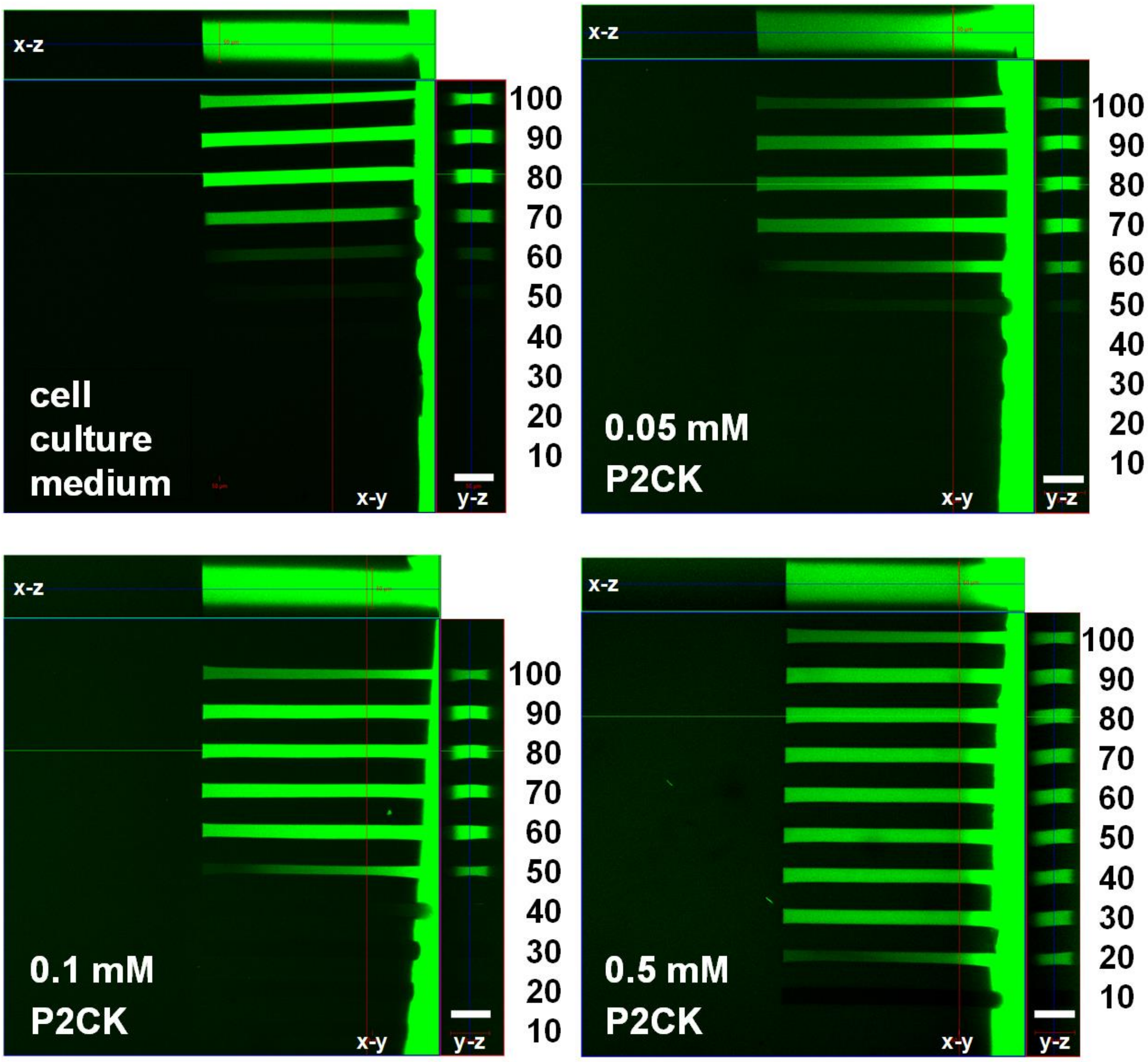

Figure S8. Micro-channel fabrication by two-photon degradation of PEG-HA-SH hydrogels in presence of two-photon sensitizer P2CK.

Top-down views of confocal z-stacks with corresponding orthogonal projections of channels (300 x 20 x 50 µm^3) fabricated at increasing laser powers (10–100 mW) in PEG-HA-SH hydrogel after swelling in different solutions of P2CK ranging from 0.05–0.5 mM or pure cell culture medium. Micro-channels were visualized by soaking in a solution of fluorescent dextran (Mw ~2,000 kDa). Scale bars = 50 µm.

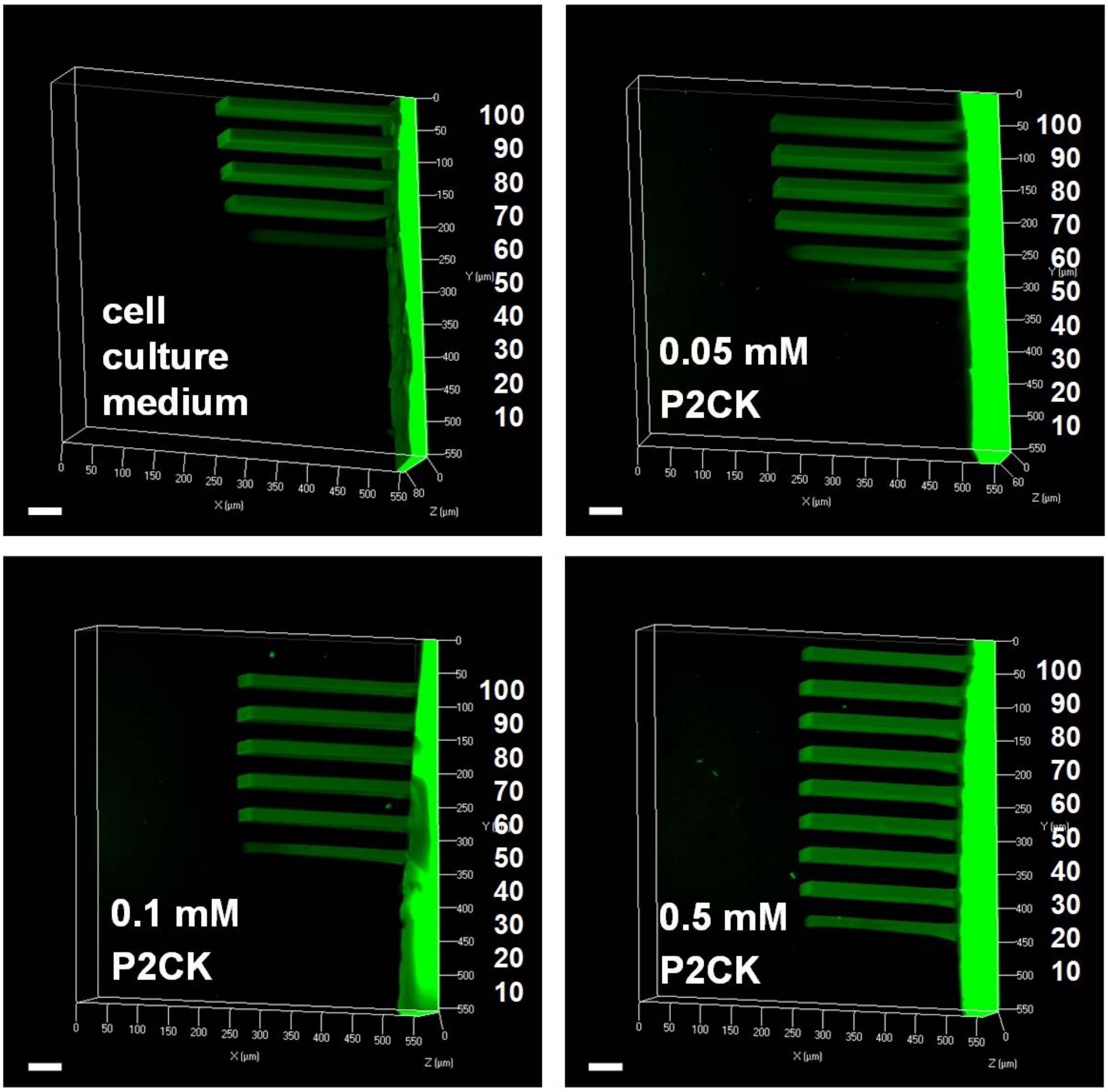

Figure S9. Micro-channel fabrication by two-photon degradation of PEG-HA-SH hydrogels in presence of two-photon sensitizer P2CK.

3D renderings of confocal z-stacks of micro-channels (300 x 20 x 50 µm^3) filled with a solution of FITC-dextran (2,000 kDa). The channels were fabricated at increasing laser powers (10–100 mW) in PEG-HA-SH hydrogel after swelling in different solutions of P2CK ranging from 0.05–0.5 mM or pure cell culture medium DMEM. Scale bars = 50 µm.

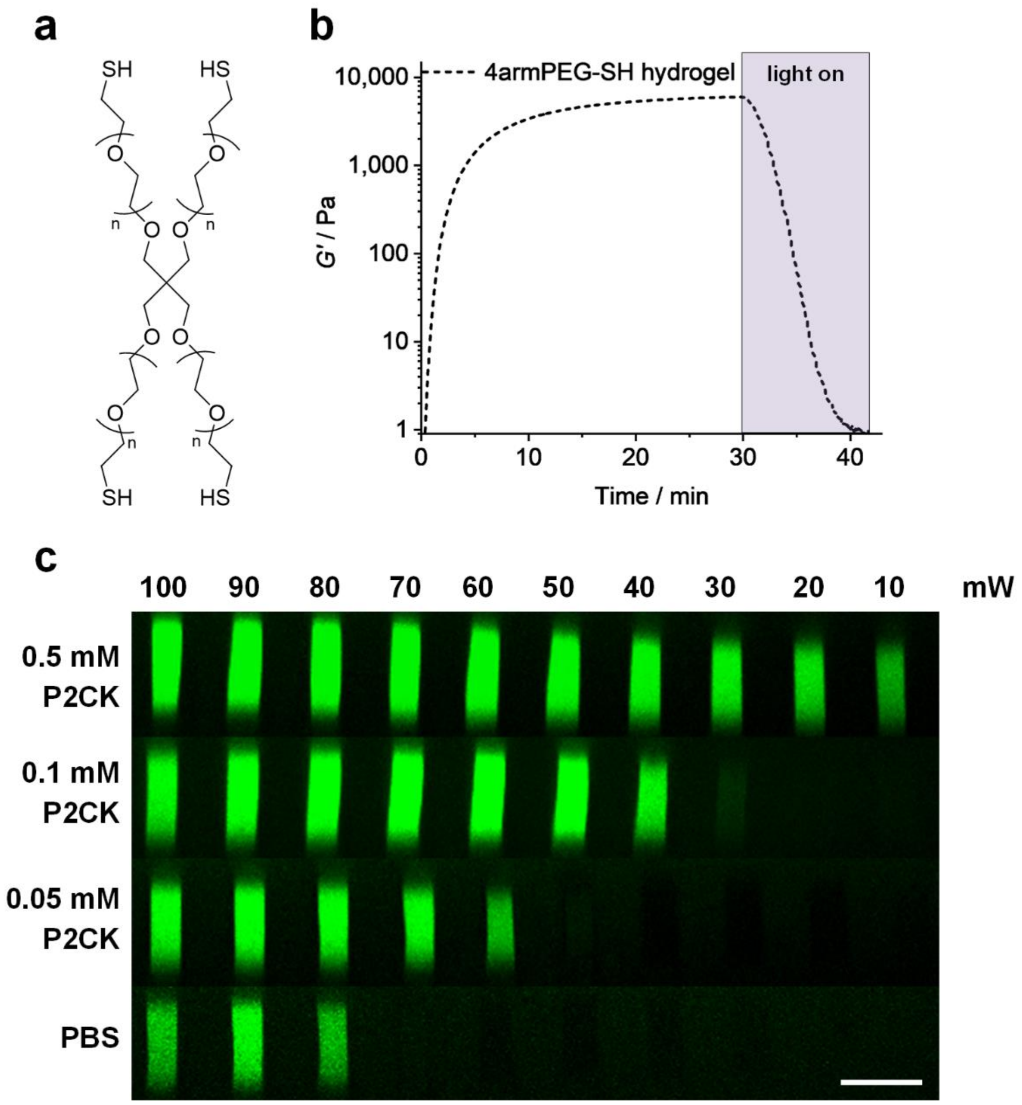

Figure S10: Formation and degradation of photodegradable 4armPEG-SH hydrogel.

a) A photodegradable hydrogel based on 4armPEG-SH and PEG-(oNB-A)$_2$ with a total macromere content of 12.5 wt% was formed by Michael-type thiol-ene reaction in a 1:1 ratio of functional groups.[4] b) Network formation and UV-VIS-induced degradation was monitored by oscillatory measurements of the shear storage modulus G'. The photodegradable 4armPEG-SH hydrogel reached an equilibrium G' of 6.5 ± 2.2 kPa (mean ± s.d.) within 30 min. Upon irradiation with UV-VIS light (320–500 nm, 20 mW cm^{-2}) the hydrogel degraded within 12 min. c) Micro-channels were fabricated by two-photon micro-patterning at varying laser powers (10–100 mW) in 4armPEG-SH hydrogels after swelling in PBS or solutions of P2CK (0.05–0.50 mM). Micro-channels were visualized by confocal microscopy using FITC-dextran. Images display orthogonal cross sections (y,z-plane). As in the case of PEG-HA-SH hydrogel the threshold laser power for two-photon erosion decreases with increasing concentration of P2CK accordingly. While in untreated hydrogel channel erosion occurs at 80 mW and above, when 0.5 mM P2CK is used the threshold can be reduced to 10 mW. Scale bar 50 µm.

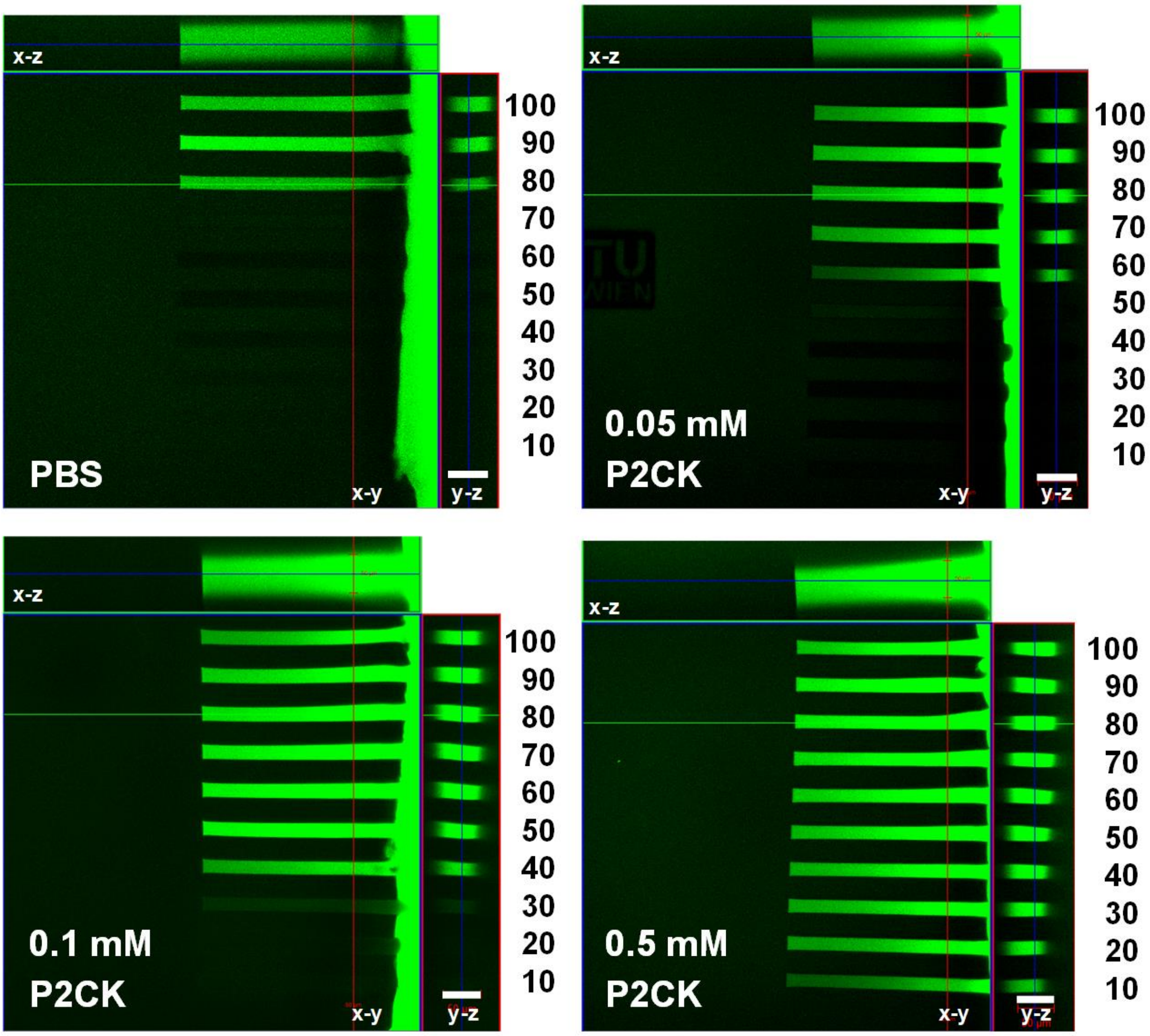

Figure S11 Micro-channel fabrication by two-photon degradation of 4armPEG-SH hydrogel in presence of two-photon sensitizer P2CK.

Top-down views of confocal z-stacks with corresponding orthogonal projections of channels (300 x 20 x 50 µm^3) fabricated at different laser powers (10–100 mW) in 4armPEG-SH hydrogel after swelling in solutions of P2CK in PBS (0.05–0.5 mM) or PBS only. Micro-channels were visualized by soaking in a solution of FITC-dextran (Mw ~2,000 kDa). Scale bars = 50 µm.

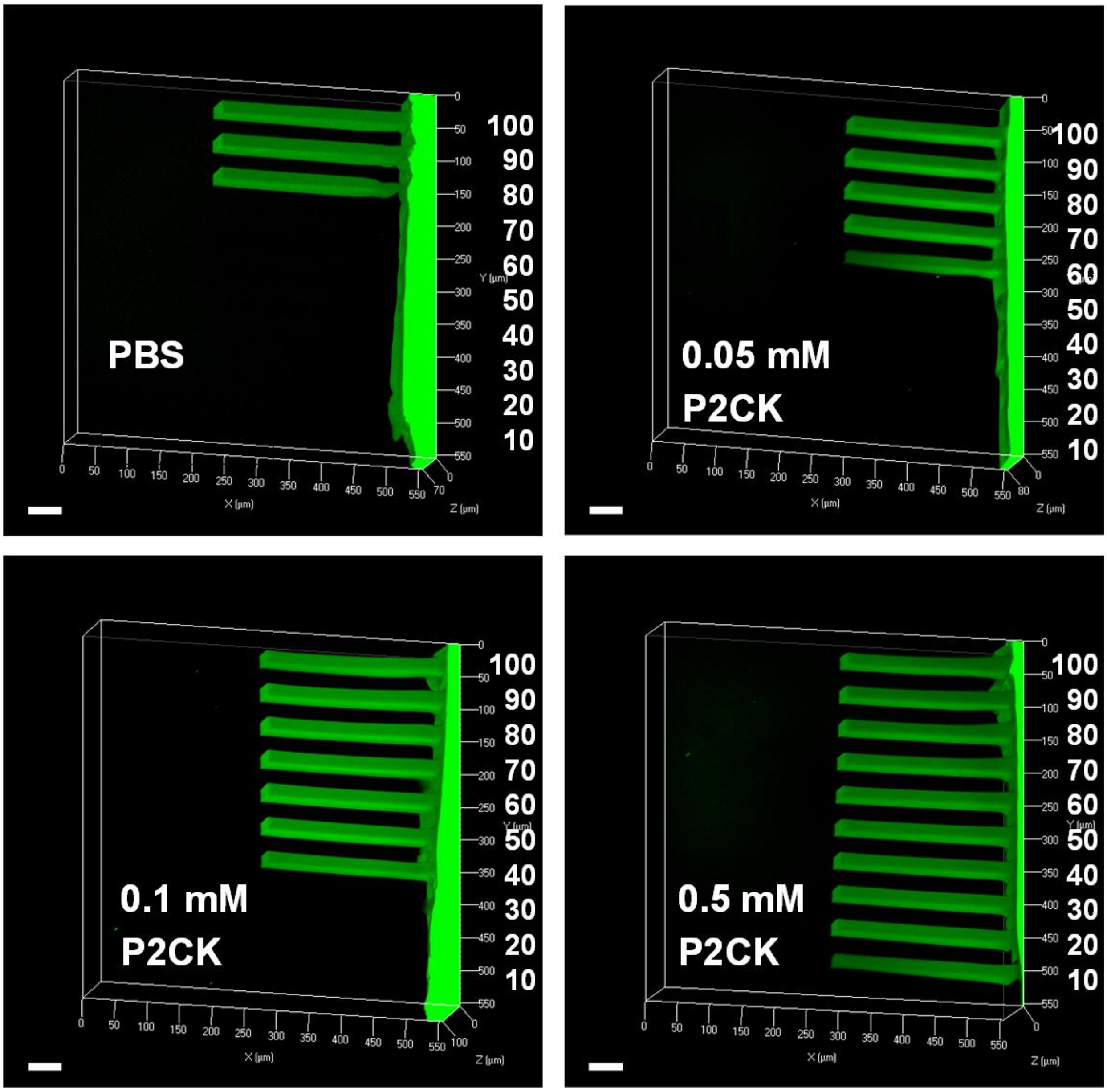

Figure S12 Micro-channel fabrication by two-photon degradation of 4armPEG-SH hydrogel in presence of two-photon sensitizer P2CK.

3D renderings of confocal z-stacks of micro-channels (300 x 20 x 50 µm^3) filled with a solution of FITC-dextran (2,000 kDa). Channels were fabricated at different laser powers (10–100 mW) in 4armPEG-SH hydrogel after swelling in solutions of P2CK in PBS (0.05–0.5 mM) or PBS only. Scale bars = 50 µm.

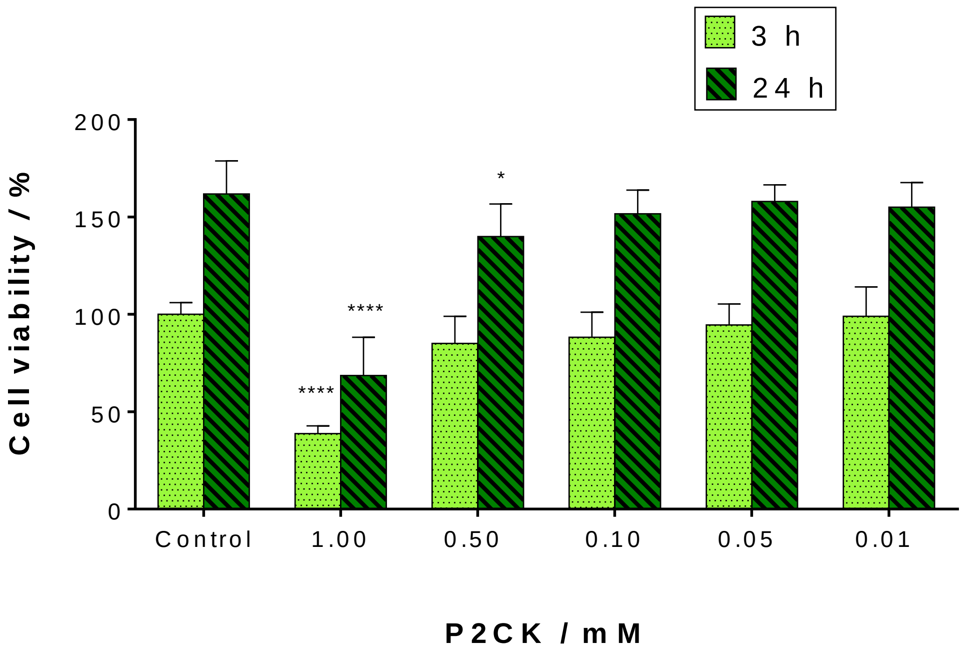

Figure S13. Effect of P2CK on ASC/TERT1.

The cell viability of ASCs exposed to P2CK for 3 h and 24 h was examined by PrestoBlue® metabolic activity assay. Data are shown as mean + standard deviation (n = 8 replicates per group) and were analyzed by one-way ANOVA followed by Dunnett's multiple comparison test. The 3 h group was compared to the 3 h control and the 24 h group was compared to the 24 h control respectively (* $P < 0.05$, **** $P < 0.0001$). Incubation of ASCs in solutions of P2CK at concentrations below 0.50 mM revealed no significant difference to the untreated control. The cell viability decreases slightly after 24 h in contact with 0.50 mM P2CK and more significantly upon incubation in 1.00 mM P2CK solution.

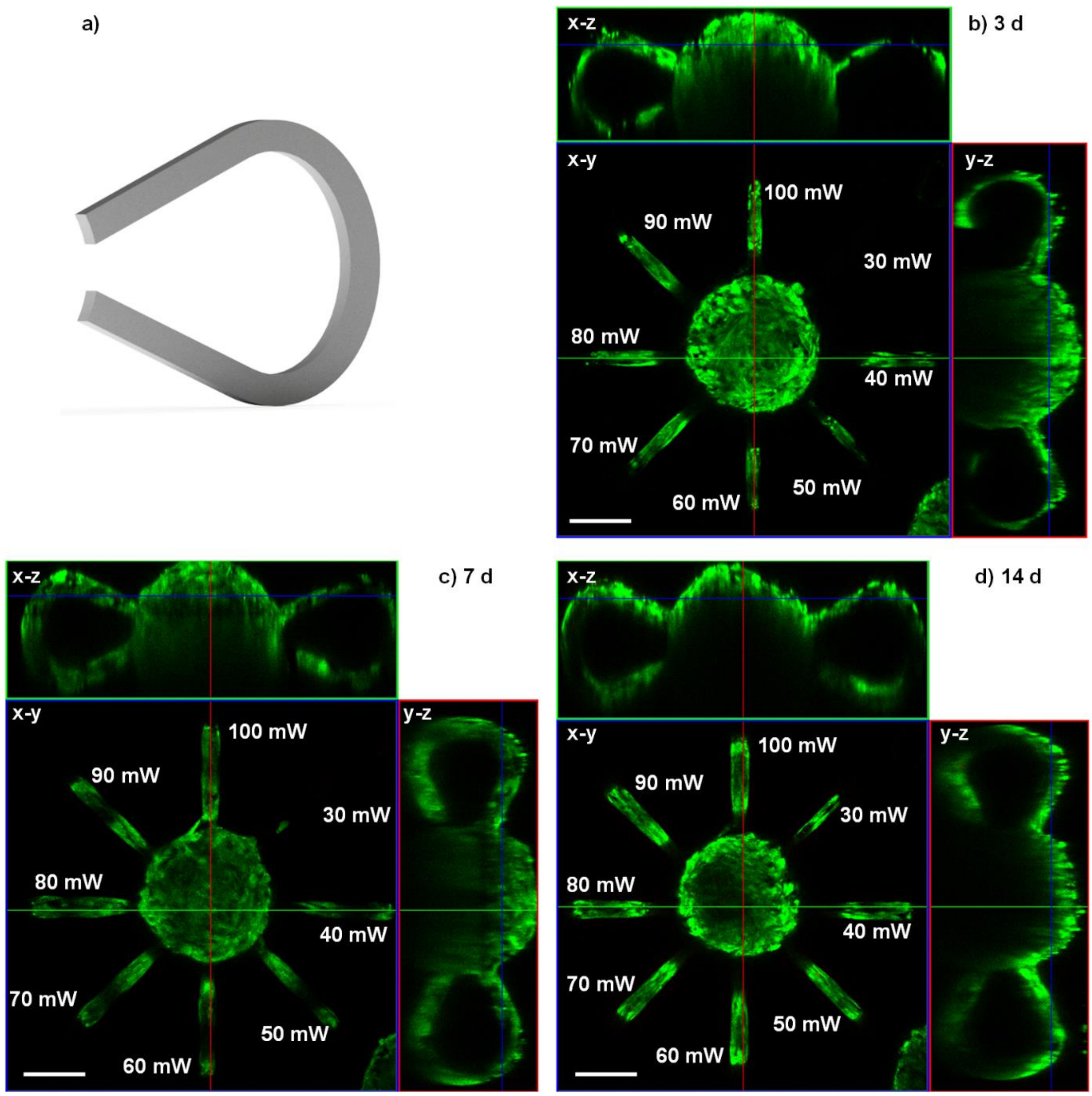

Figure S14. ASC/TERT1 spreading into loops micropatterned with P2CK.

a) 3D model of a horseshoe-shaped channel (Ø = 20 µm x 20 µm) generated by CAD. 24 h after spheroid encapsulation the hydrogel was soaked in a solution of P2CK (0.1 mM) for 5.5 h and then horseshoe-shaped channels (Ø = 20 µm x 20 µm) were micropatterned from spheroids into the bulk hydrogel at different laser powers ranging from 30–100 mW. b) Within 3 days after micropatterning, cells had entered loops fabricated at laser powers down to 40 mW, indicating that at these conditions the network in the irradiated areas had been sufficiently disintegrated for cells to penetrate. c) While the area irradiated at 30 mW remained unoccupied for 7 days, d) cells filled up this loop after 14 days, most likely due to ester hydrolysis of remaining linkages. c,d) Excellent cell viability is indicated by high GFP- (green) and very low PI-signal (red). Scale bars 100 µm.

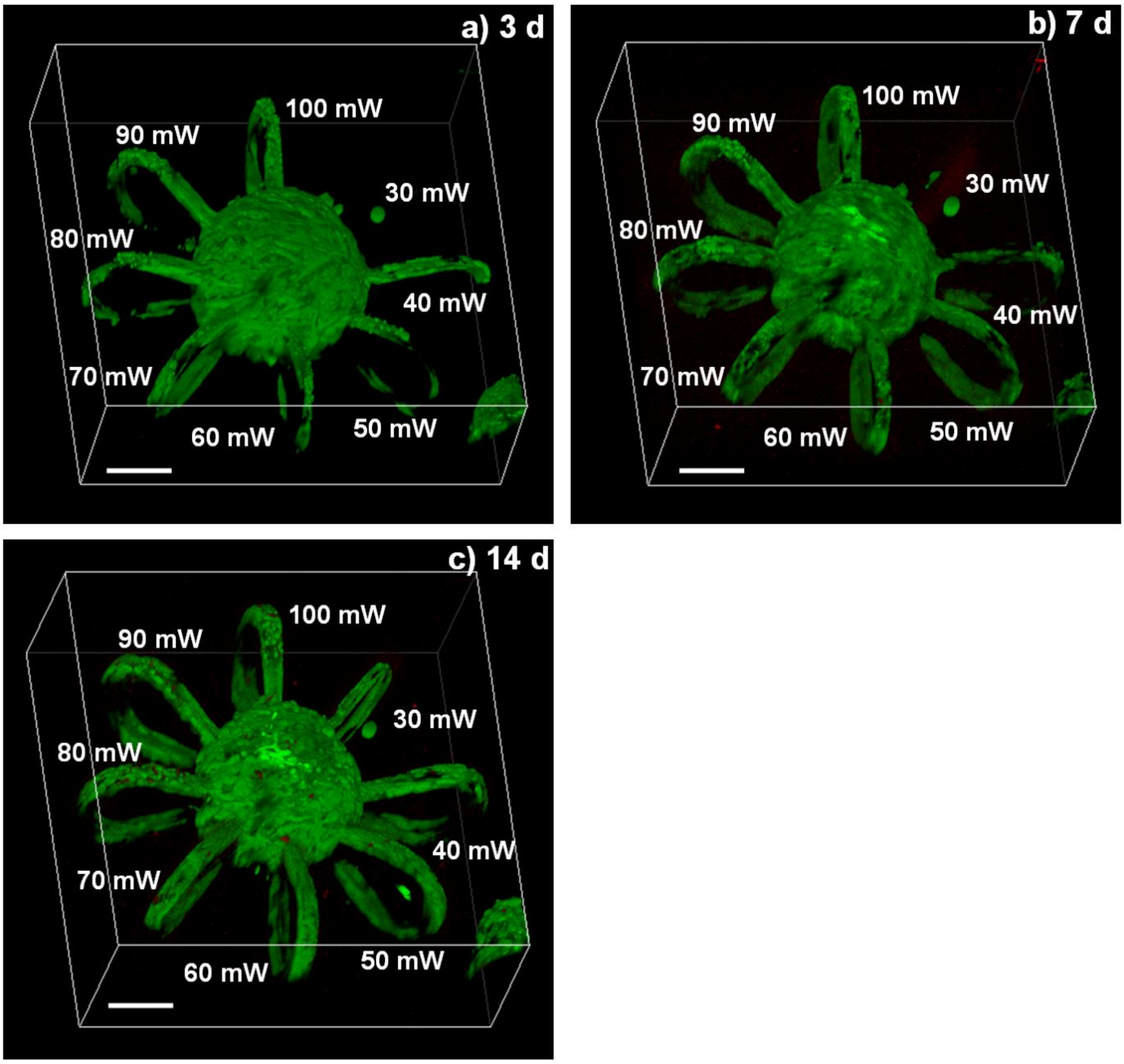

Figure S15. ASC/TERT1 spreading into loops micropatterned with P2CK.

3D renderings of confocal z-stacks of GFP-labeled ASCs spreading into horseshoe-shaped channels (Ø = 20 µm x 20 µm). Loops were micropatterned in presence of P2CK (0.1 mM) at laser powers ranging from 30–100 mW. a) Within 3 days after micropatterning, cells had entered loops fabricated at laser powers down to 40 mW, indicating that at these conditions the network in the irradiated areas had been sufficiently disintegrated for cells to penetrate. b) While the area irradiated at 30 mW remained unoccupied for 7 days, c) cells filled up this loop after 14 days, most likely due to ester hydrolysis of remaining linkages. b,c) Excellent cell viability is indicated by high GFP- (green) and very low PI-signal (red). Scale bars 100 µm.

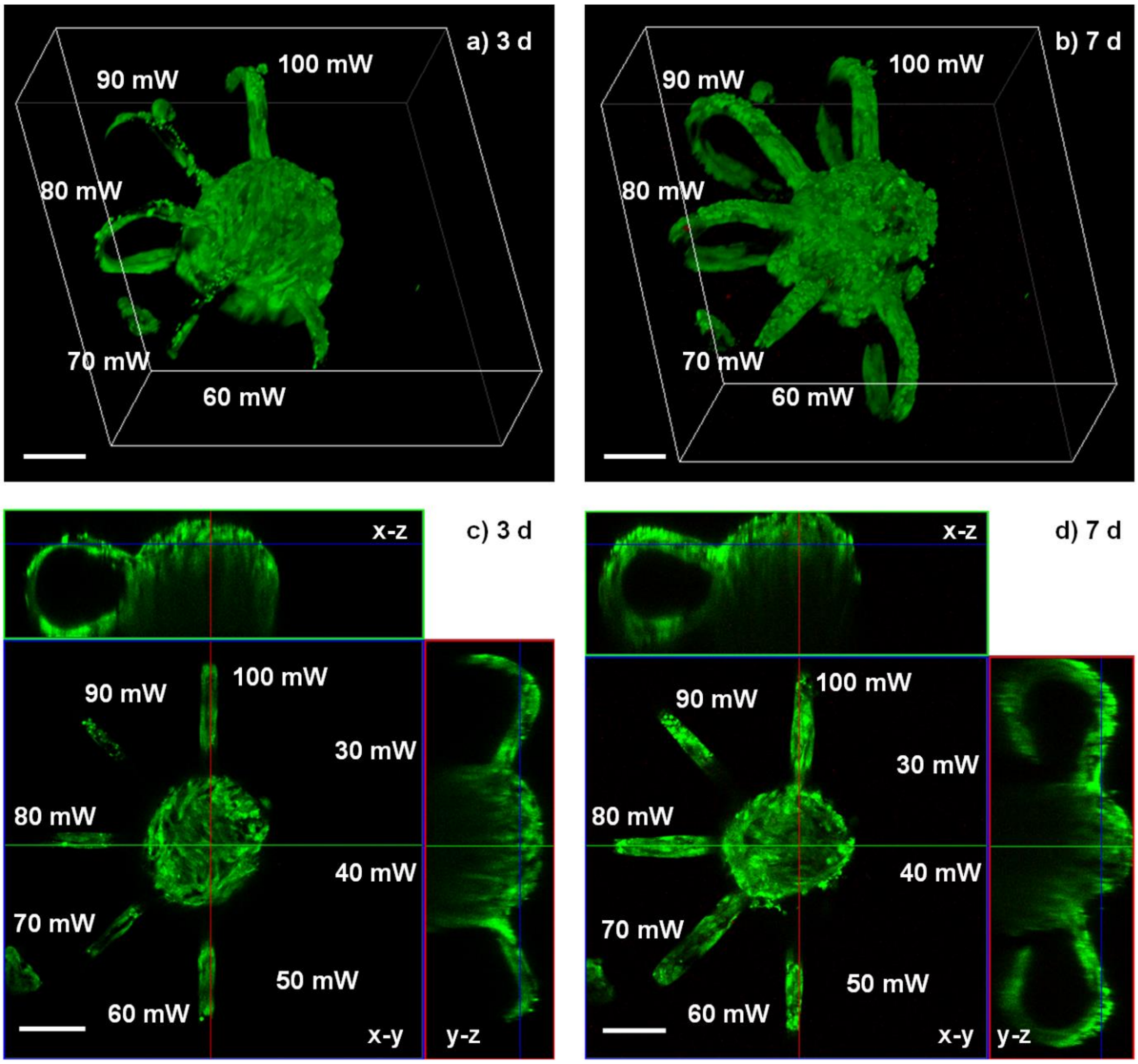

Figure S16. ASC/TERT1 spreading into loops micropatterned without P2CK.

In a control experiment, horseshoe-shaped channels were micropatterned around a spheroid at different laser powers (30–100 mW) in the absence of P2CK. a, c) Within 3 days after two-photon micropatterning cells had only partially entered channels fabricated at laser powers of 60 mW and above. b,d) Cells did not spread into channels fabricated at lower laser power even after 7 days. Excellent cell viability is indicated by high GFP- (green) and very low PI-signal (red) after 7 days. Scale bars 100 µm.

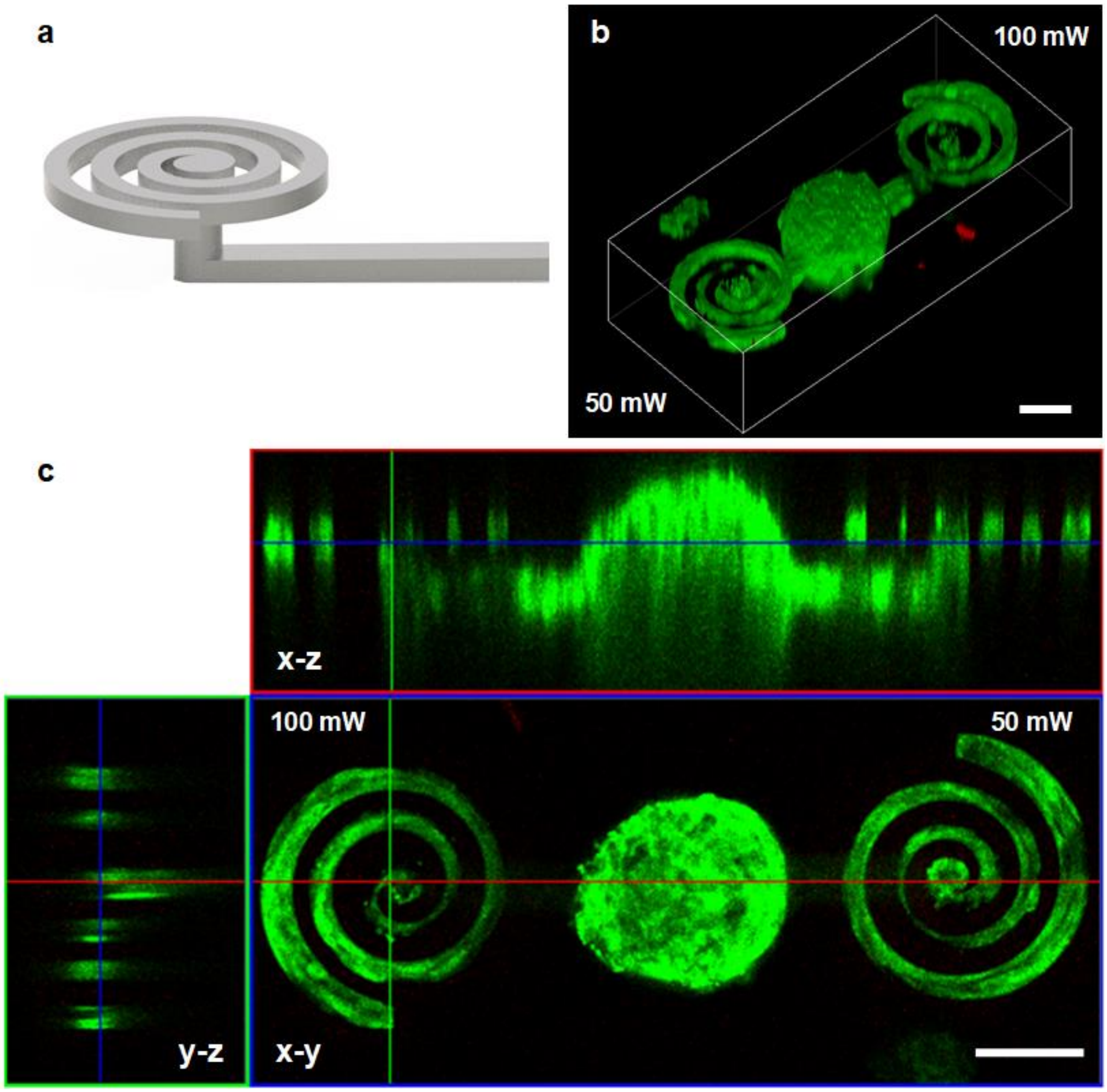

Figure S17. ASC/TERT1 spreading into two-photon micropatterned spirals.

a) A complex 3D-object consisting of a spiral which is connected to a channel in another z-plane was generated by CAD. Spirals were produced at 50 mW and 100 mW in presence of P2CK (0.10 mM) in a way that they were connected to a spheroid via the channel. After 7 d cells filled up both spirals as visualized by b) 3D renderings and c) orthogonal projections of confocal z-stacks. High GFP- (green) and very low PI-signal (red) indicate excellent cell viability. Scale bars = 100 µm.

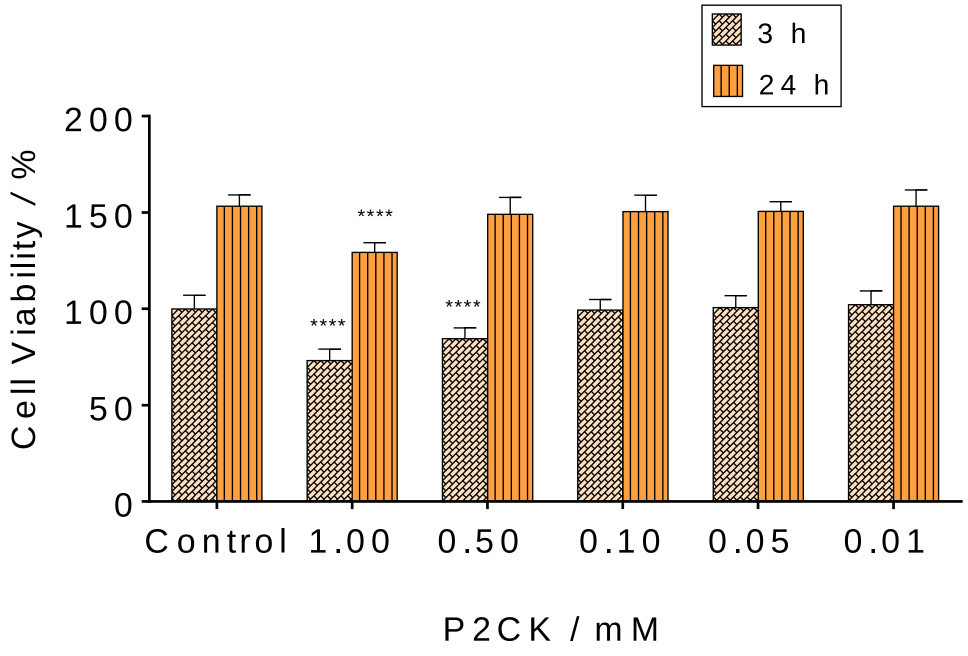

Figure S18. Effect of P2CK on human osteosarcoma cells (MG-63).

The cell viability of MG-63s exposed to P2CK for 3 h and 24 h was examined by PrestoBlue® metabolic activity assay. Data are shown as mean + standard deviation (n = 8 replicates per group) and were analyzed by one-way ANOVA followed by Dunnett's multiple comparison test. The 3 h group was compared to 3 h control and the 24 h group was compared to the 24 h control (* $P < 0.05$, **** $P < 0.0001$). Incubation of MG-63s in solutions of P2CK at concentrations below 0.50 mM revealed no significant difference to the untreated control. The cell viability decreases significantly after 3 h in contact with 0.50 mM P2CK and more significantly upon incubation in 1.00 mM P2CK solution.

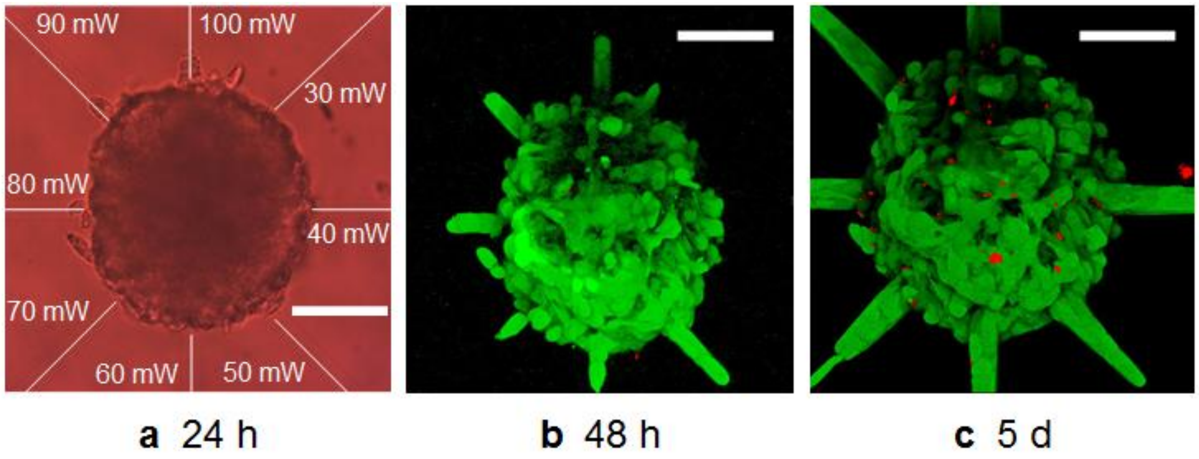

Figure S19. Spreading of human osteosarcoma cells (MG-63) into channels two-photon micropatterned in PEG-HA-SH hydrogel.

a) Light microscopy image of a MG-63 spheroid encapsulated in PEG-HA-SH hydrogel (DS = 40%). Linear micro-channels (300 µm x 20 µm x 20 µm) were eroded from the spheroid into the bulk hydrogel with increasing laser powers ranging from 30–100 mW in a clockwise manner, after swelling the hydrogel for 1 h in a 0.1 mM P2CK solution. The light microscopy image was taken 24 h after the channels were produced. Micro-channels are indicated by thin white lines. LIVE/DEAD staining was performed to investigate both the viability and spreading of MG-63 cells into the channels after b) 48 h and c) 5 days showing high cell viability. Green: calcein AM; Red: PI; Scale bars = 100 µm.

S3 References

[1] X. Z. Shu, Y. Liu, Y. Luo, M. C. Roberts, G. D. Prestwich, *Biomacromolecules* **2002**, *3*, 1304-1311.

[2] A. M. Kloxin, M. W. Tibbitt, K. S. Anseth, *Nat. Protoc.* **2010**, *5*, 1867-1887.

[3] Z. Li, J. Torgersen, A. Ajami, S. Mühleder, X. Qin, W. Husinsky, W. Holnthoner, A. Ovsianikov, J. Stampfl, R. Liska, *RSC Adv.* **2013**, *3*, 15939-15946.

[4] M. W. Tibbitt, A. M. Kloxin, L. A. Sawicki, K. S. Anseth, *Macromolecules* **2013**, *46*, 2785-2792.

[5] C. Gorsche, R. Harikrishna, S. Baudis, P. Knaack, B. Husar, J. Laeuger, H. Hoffmann, R. Liska, *Anal. Chem.* **2017**, *89*, 4958-4968.

[6] A. Ovsianikov, S. Mühleder, J. Torgersen, Z. Li, X.-H. Qin, S. Van Vlierberghe, P. Dubruel, W. Holnthoner, H. Redl, R. Liska, J. Stampfl, *Langmuir* **2014**, *30*, 3787-3794.

[7] J. L. Hutter, J. Bechhoefer, *Rev. Sci. Instrum.* **1993**, *64*, 1868-1873.

[8] L. Kain, O. G. Andriotis, P. Gruber, M. Frank, M. Markovic, D. Grech, V. Nedelkovski, M. Stolz, A. Ovsianikov, P. J. Thurner, *Journal of the Mechanical Behavior of Biomedical Materials* **2018**, *85*, 225-236.

[9] C. Neto, V. S. J. Craig, *Langmuir* **2001**, *17*, 2097-2099.

[10] L. Knezevic, M. Schaupper, S. Mühleder, K. Schimek, T. Hasenberg, U. Marx, E. Priglinger, H. Redl, W. Holnthoner, *Front. Bioeng. Biotechnol.* **2017**, *5,* 25.

[11] W. R. Zipfel, R. M. Williams, W. W. Webb, *Nat. Biotechnol.* **2003**, *21*, 1369-1377.

[12] C. Heller, N. Pucher, B. Seidl, K. Kalinyaprak-Icten, G. Ullrich, L. Kuna, V. Satzinger, V. Schmidt, H. C. Lichtenegger, J. Stampfl, R. Liska, *J. Polym. Sci., Part A: Polym. Chem.* **2007**, *45*, 3280-3291.
